# Autoimmunity in Arabidopsis *acd11* Is Mediated by Epigenetic Regulation of an Immune Receptor

**DOI:** 10.1371/journal.ppat.1001137

**Published:** 2010-10-07

**Authors:** Kristoffer Palma, Stephan Thorgrimsen, Frederikke Gro Malinovsky, Berthe Katrine Fiil, H. Bjørn Nielsen, Peter Brodersen, Daniel Hofius, Morten Petersen, John Mundy

**Affiliations:** 1 Department of Biology, University of Copenhagen, Copenhagen, Denmark; 2 Department of Systems Biology, Technical University of Denmark, Kongens Lyngby, Denmark; The University of North Carolina at Chapel Hill, United States of America

## Abstract

Certain pathogens deliver effectors into plant cells to modify host protein targets and thereby suppress immunity. These target modifications can be detected by intracellular immune receptors, or Resistance (R) proteins, that trigger strong immune responses including localized host cell death. The *accelerated cell death 11* (*acd11*) “lesion mimic” mutant of *Arabidopsis thaliana* exhibits autoimmune phenotypes such as constitutive defense responses and cell death without pathogen perception. *ACD11* encodes a putative sphingosine transfer protein, but its precise role during these processes is unknown. In a screen for *lazarus* (*laz*) mutants that suppress *acd11* death we identified two genes, *LAZ2* and *LAZ5*. *LAZ2* encodes the histone lysine methyltransferase SDG8, previously shown to epigenetically regulate flowering time via modification of histone 3 (H3). *LAZ5* encodes an RPS4-like R-protein, defined by several dominant negative alleles. Microarray and chromatin immunoprecipitation analyses showed that LAZ2/SDG8 is required for *LAZ5* expression and H3 lysine 36 trimethylation at *LAZ5* chromatin to maintain a transcriptionally active state. We hypothesize that LAZ5 triggers cell death in the absence of ACD11, and that cell death in other lesion mimic mutants may also be caused by inappropriate activation of *R* genes. Moreover, SDG8 is required for basal and R protein-mediated pathogen resistance in Arabidopsis, revealing the importance of chromatin remodeling as a key process in plant innate immunity.

## Author Summary

Plants defend themselves against pathogens via immune receptors that trigger responses including the suicide of infected cells to limit pathogen growth. The *accelerated cell death 11* (*acd11*) knockout mutant of the model plant *Arabidopsis thaliana* kills itself in the absence of invading pathogens. By screening for secondary mutations that resurrect *acd11*, we discovered two *LAZARUS* (*LAZ*) genes required for death. The first, *LAZ2*, encodes an enzyme that methylates histones, the major protein component of chromatin. This particular histone modification is generally involved in epigenetic remodeling of chromatin to a more permissive state for transcription of associated DNA. We show that expression of the second gene, *LAZ5*, is dependent on LAZ2 activity, suggesting that LAZ5 is a direct target of LAZ2. LAZ5 is a member of an immune receptor class involved in detection of specific pathogens and subsequent cell death. We propose that *acd11*, and other suicidal mutants, result from autoimmunity triggered by immune receptors controlled by chromosomal modifications. Interestingly, we found that defects in LAZ2 result in enhanced susceptibility to bacterial pathogens, suggesting that it controls other genes involved in innate immunity.

## Introduction

Unlike vertebrates, plants lack a somatic, adaptive immune system and immunological memory [1]. Therefore, plants rely on a large repertoire of pre-existing immune receptors, encoded by hypervariable *Resistance* (*R*) genes, which recognize specific pathogens and activate strong defense responses. These responses include the programmed cell death (PCD) of host cells at infection sites to restrict pathogen access in a process called the hypersensitive response (HR). R proteins are triggered by pathogen-specific effector proteins that have evolved to perturb or disrupt host processes to facilitate infection. While some pathogen effectors are recognized extracellularly, the majority are targeted to various intracellular compartments of the plant host and identified there. In most cases, R proteins are activated by detecting modifications to host proteins targeted by pathogen effectors. This model, known as the “guard hypothesis” [2], [3], has been supported in numerous instances. For example RIN4, a host protein with key roles in basal defense, is under surveillance by multiple R proteins, and at the same time is the target of multiple pathogen effectors [4]. Most R proteins have been classified as NB-LRRs, named after their central nucleotide-binding (NB) and C-terminal leucine-rich repeat (LRR) domains, although various exceptions exist [5]. The N-terminal domains of NB-LRR R proteins fall into two broad categories: those with homology to *Drosophila* Toll and mammalian Interleukin-1 Receptor (TIR), and those with predicted coiled-coil (CC) regions [6]. Members of the animal NOD-like receptor (NLR) family exhibit similar domain architecture to plant NB-LRRs, and NLRs are likewise involved in immunity [7], [8]. Like NB-LRR proteins, NLRs have several types of amino-termini including protein–protein interaction domains associated with proteins involved in programmed cell death and inflammation. Several autoimmune diseases in humans have been associated with mutations in NLRs [9].

In plants, there are numerous examples of mutants with autoimmunity-related phenotypes. These so-called “lesion-mimics” are, in many cases, caused by mutations in genes hypothesized to be negative regulators of the HR [10]. Other examples include point mutations in NB-LRR R proteins [11], [12]. Since R proteins have the potential to trigger host PCD, their activity is tightly regulated. *R* genes are typically constitutively expressed at low levels and some are up-regulated in response to pathogen-derived peptides or to the accumulation of the phytohormone salicylic acid (SA) [13], [14]. Little is known about the transcriptional control of *R* genes. Intriguingly, members of a cluster of related *Arabidopsis R* genes are endogenously suppressed at the post-transcriptional level by RNA silencing, suggesting that pathogens that interfere with the silencing machinery unwittingly up-regulate steady-state R protein levels [15]. At the protein level, inappropriate activation is likely prevented by autoinhibition, high rates of turnover, and alternatively spliced products. Recently, it has become clear that hybrid necrosis, a deleterious genetic incompatibility observed in many intra- and interspecific plant hybrids, is associated with autoimmunity [16]. One example of this type of autoimmune response in *Arabidopsis* was shown to be dependent on an NB-LRR R protein, suggesting that these immune receptors have a broad mandate over PCD that extends beyond pathogen defense [17].

The lethal, recessive *accelerated cell death 11* (*acd11*) mutant of *Arabidopsis* is characterized by constitutive activation of immune responses and PCD in the absence of pathogen attack [18]. *ACD11* encodes a putative sphingosine transfer protein with homology to HET-C2 of the fungus *Podospora anserina*. Allelic variants of *het-c* determine compatibility during fusion of hyphae from different strains, causing PCD in combination with specific alleles at other *het* loci [19]. *acd11* mutants develop normally until the 2–4 leaf stage, and PCD involves the phytohormone SA such that expression of a bacterial SA hydroxylase (NahG) strongly suppresses cell death. Application of SA agonists, such as benzothiadiazol-*S*-methyl ester (BTH), restores autoimmunity in *acd11*. Interestingly, the genetic requirements for *acd11* cell death are similar to those for the HR triggered by TIR-NB-LRR immune receptors [18], [20].

We report here that cell death in *acd11* is suppressed by mutations in genes encoding a histone methyltransferase and a TIR-NB-LRR R protein. In addition, the expression of the *R* gene is dependent on the activity of the histone modifying enzyme. We propose that the TIR-NB-LRR is triggered by the absence of ACD11, implying that ACD11 (or a complex containing ACD11) may be a guarded pathogen effector target. Alternatively, since ACD11 may be involved in production of a lipid signal, the absence of this signal may induce *LAZ5* expression in an SA-dependent manner. Our study provides strong evidence that a specific type of histone modification is directly involved in chromatin remodeling and transcriptional control of a subset of *R* genes including *LAZ5*.

## Results

### *laz2* suppresses cell death in *acd11*

**Figure 1:**
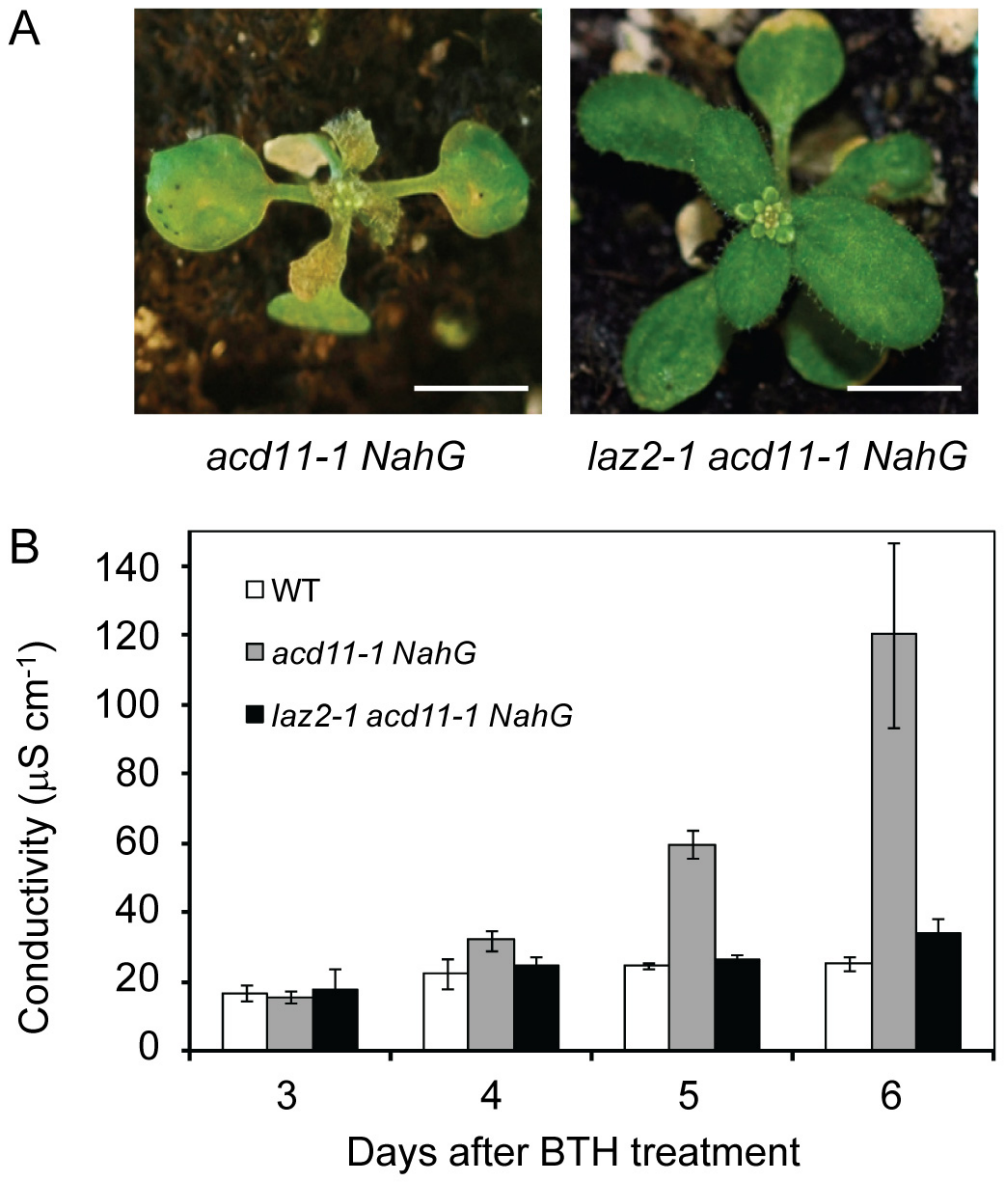
*laz2* suppresses cell death in *acd11*. **A**, 21-day-old *acd11-1 NahG* and *laz2-1 acd11-1 NahG* plants 1 week after treatment with 100 µM BTH. Size bar = 0.5 cm. **B**, Ion leakage cell death assay of leaf discs from 5-week-old L*er* wild-type (WT), *acd11-1 NahG* and *laz2-1 acd11-1 NahG* plants after BTH treatment. Means ± s.d. were calculated from 4 discs per treatment with 4 replicates within an experiment.

To isolate genes required for cell death in *acd11*, Landsberg *erecta* (L*er*) ecotype *acd11-1* plants harboring the *NahG* transgene were mutagenized with ethyl-methanesulfonate (EMS), diepoxybutane (DEB) or γ-irradiation. ∼200 suppressors of *acd11* were subsequently identified as plants that survived following BTH treatment. Genetic analyses of 43 such suppressors grouped them into 12 recessive and 2 dominant loci referred to as *lazarus* (*laz*) mutants, after the biblical resurrection. One of the *laz* mutants found in the suppressor screen, *laz2*, abolished cell death in response to BTH in the *acd11 NahG* background, and exhibited similar levels of cellular ion leakage as wild type (Fig. 1, A and B). *laz2-1 acd11-1 NahG* plants also exhibited abnormal development (e.g. early flowering, increased shoot branching) that, along with *acd11* suppression, was inherited recessively (data not shown). Two other *laz2* alleles with similar morphology, *laz2-2* and *laz2-3*, were confirmed by complementation tests (Fig. S1A). Global transcript profiles of *laz2-1 acd11-1 NahG*, L*er* wild-type, *NahG*, and *acd11-1 NahG* plants were acquired by hybridizing total mRNA, isolated before and 72 h after BTH treatment, to Affymetrix ATH1 GeneChip arrays. *laz2-1* exhibited dramatic suppression of the top 500 most significantly regulated genes in *acd11-1* after 72 h BTH (Fig. S2A) In addition, a strong negative Pearson correlation of −0.87 was obtained for global expression fold change between *laz2-1 acd11-1* and *acd11-1*, indicating that gene expression in *acd11-1* was strongly affected by the *laz2-1* mutation (Fig. S2B).

**Figure 2:**
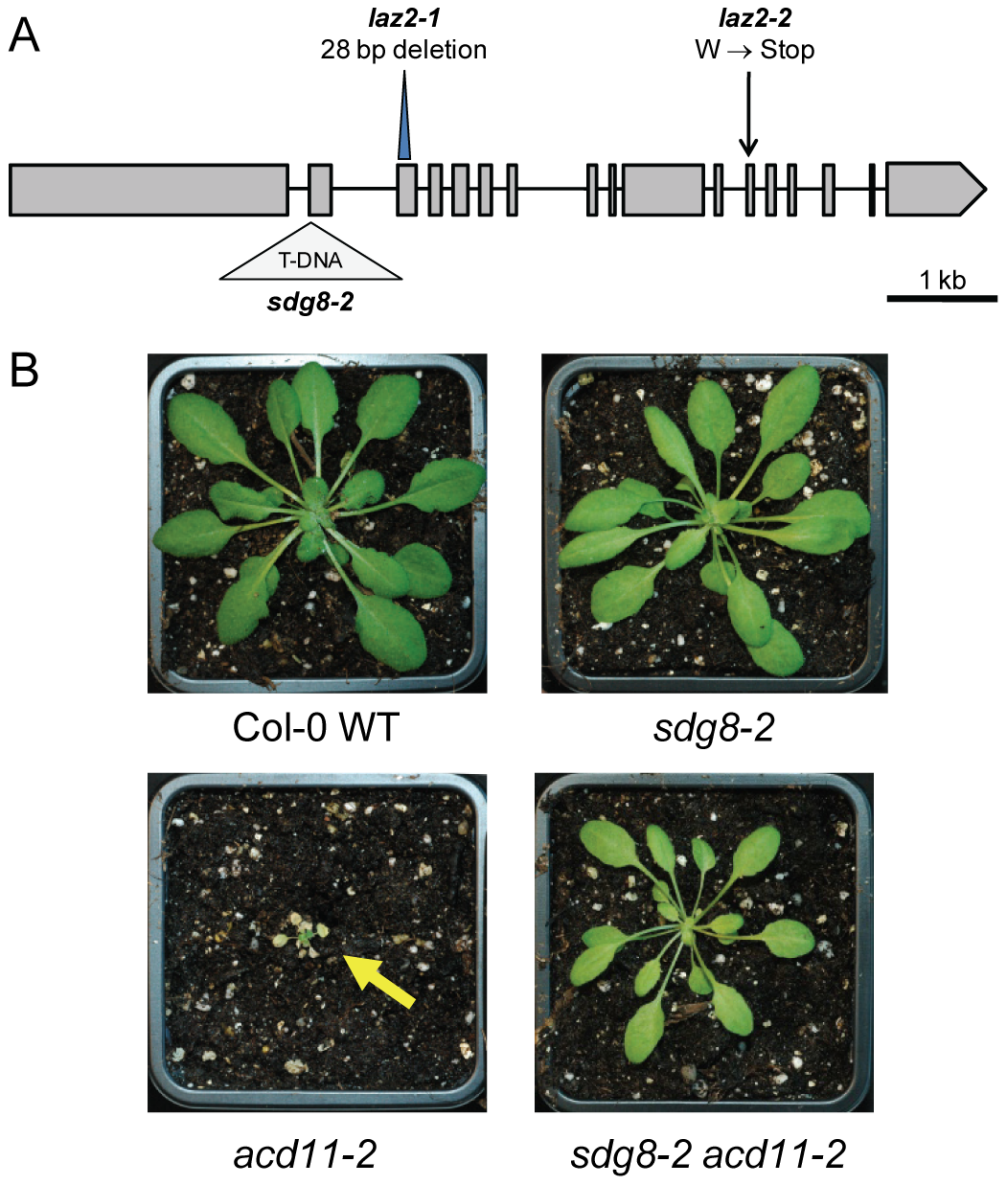
*LAZ2* encodes a histone lysine methyltransferase that is essential for cell death in *acd11*. **A**, *SDG8* gene with locations of the *laz2-1* deletion, *laz2-2* mutation, and *sdg8-2* T-DNA insertion (SALK_026642). Boxes and lines represent exons and introns, respectively. 1 kb = 1000 base pairs. **B**, Col-0 WT, *sdg8-2*, *acd11-2* and *sdg8-2 acd11-2* plants 4 weeks after germination.

The *LAZ2* locus was identified using a map-based approach. Briefly, L*er laz2-1 acd11 NahG* was crossed to Columbia ecotype (Col-0) *acd11 NahG* to generate a segregating F_2_ mapping population after BTH treatment. Ecotype-specific linkage markers were used to map *laz2-1* to a ∼150 kb region at the bottom of chromosome 1 (Fig. S3). Candidate genes were selected and sequenced based on annotated mutant phenotypes at The *Arabidopsis* Information Resource (TAIR; http://www.arabidopsis.org), revealing an irradiation-induced 28-bp deletion in the third exon of the gene *At1g77300* (Fig. 2A). This locus was also sequenced in *laz2-2 acd11-1 NahG*, revealing an EMS-induced G to A transition converting tryptophan 1536 to a premature stop.

### *LAZ2* encodes the histone methyltransferase SDG8

Sequence analysis revealed that *LAZ2* encodes the histone lysine methyltransferase (HKMT) SET (Su(var)3-9, E(z) and Trithorax-conserved) DOMAIN GROUP 8 (SDG8), otherwise known as EARLY FLOWERING IN SHORT DAYS (EFS) and CAROTENOID CHLOROPLAST REGULATORY 1 (CCR1) [21], [22]. The mutation in *laz2-1* causes a frame-shift just upstream of the sequence encoding the conserved SET associated cysteine-rich domains, while that in *laz2-2* introduces a stop codon upstream of a motif conserved within the RPB1 subunits of RNA polymerase II [23]. SDG8 is homologous to yeast SET2, which is associated with methylations at histone 3 lysine 36 (H3K36). Another yeast HKMT, SET1, modifies H3K4. Both H3K4 and H3K36 methylation marks are typically associated with active transcription [24]. While *Arabidopsis* has 43 annotated SDG proteins, SDG8 groups with H3K36-specific HKMTs in fungi and animals along with 4 other *Arabidopsis* proteins [25]. During transcription in yeast, SET1 and SET2 are recruited to active chromatin by the RNA polymerase II-associated PAF1 complex, where they promote gene expression by facilitating chromatin opening, thus enhancing transcription initiation and elongation, respectively [26]. A similar mechanism seems to be conserved in *Arabidopsis* based on studies of *sdg* mutants. *SDG8* was first identified as a gene that controlled flowering time via its activity on the transcription of the key floral repressor *FLOWERING LOCUS C* (*FLC*), an epigenetically regulated MADS box transcription factor (TF) [27], [28]. Expression of the *FLC* paralog *MADS AFFECTING FLOWERING 1* (*MAF1*) is also dependent on SDG8, which is required for di- and trimethylation of H3K36 [25]. In addition to flowering time, SDG8 regulates carotenoid composition and shoot branching via modification of chromatin at specific loci [22], [29]. Our microarray expression analysis revealed that *MAF1* and *CRTISO*, both recently confirmed as direct targets of SDG8 [22], [25], exhibited very low expression levels in the absence of *LAZ2* (Fig. S4). Deficient expression of these and similar genes likely contributes to the developmental phenotypes observed in *laz2*. Furthermore, the loss-of-function mutant *sdg8-2* (SALK_026642) shared *laz2* morphology (Fig. S1B) and suppressed *acd11-2*, an *ACD11* knockout in the Col-0 ecotype (Fig. 2B).

### Cell death in *acd11* is dependent on the *R* gene LAZARUS 5

**Figure 3:**
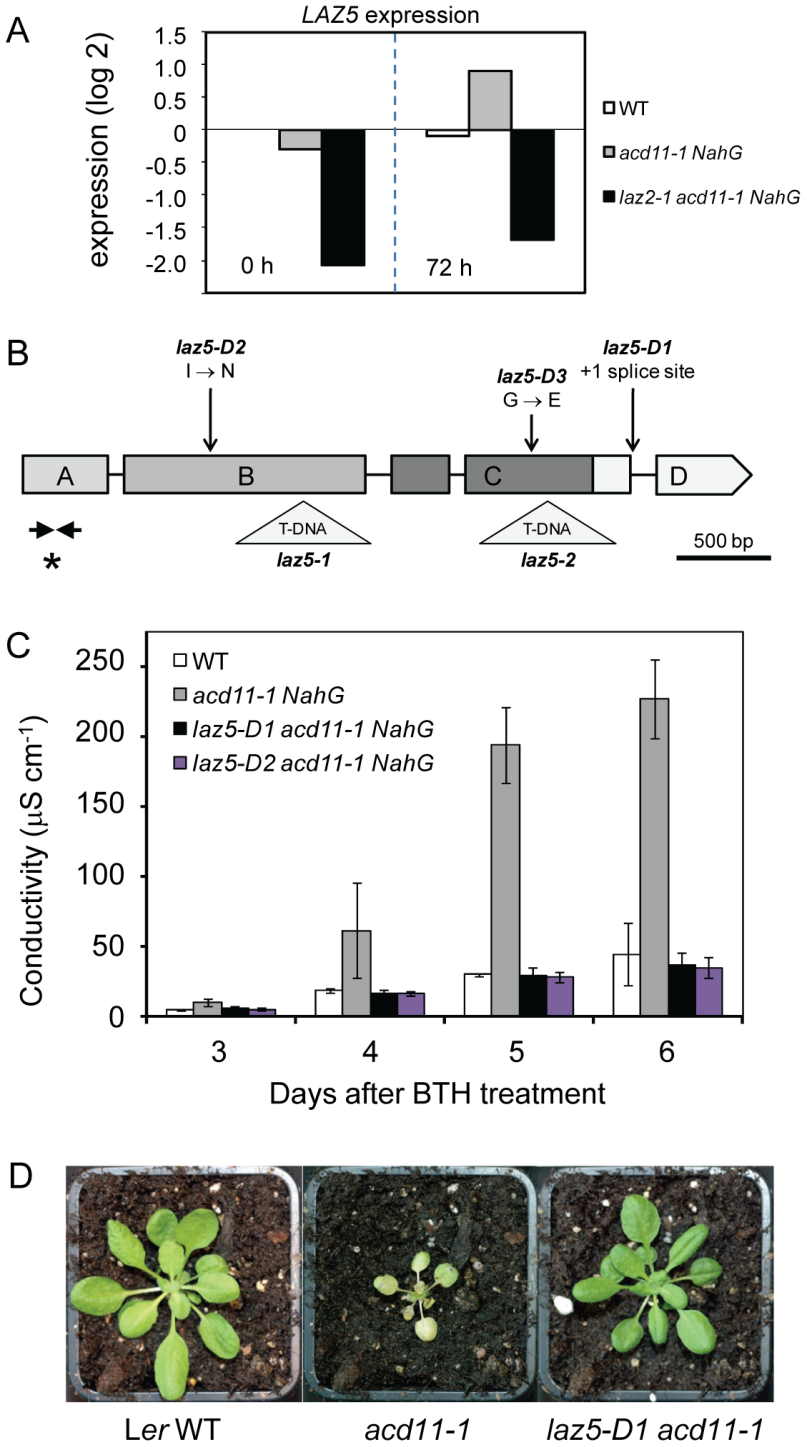
*acd11* autoimmunity requires LAZ5, a TIR-NB-LRR immune receptor. **A**, Expression of *At5g44870* (*LAZ5*) in L*er* WT, *acd11-1 NahG* and *laz2-1 acd11-1 NahG* before and 72 h after treatment with 100 µM BTH relative to L*er* WT at time 0 (log2 scale). Microarrays were performed on duplicates or triplicates of independent RNA preparations from aerial parts of 4-week-old plants before and 72 h after BTH treatment. *LAZ5* is significantly differentially expressed between the genotypes (p = 7e-7) and over time (p = 0.005) as determined by Two-Way ANOVA. **B**, *LAZ5* with locations of mutations in 3 *laz5-D* alleles and the 2 T-DNA insertions *laz5-1* (SALK_087262) and *laz5-2* (SAIL_874-D10). Boxes and lines represent exons and introns. Domains encoded by exons are marked TIR (A), NB (B), LRR (C), C-terminal extension (D). Asterisk marks the region amplified for ChIP and short arrows represent flanking primers. 500 bp = base pairs. **C**, Ion leakage death assay of leaf discs from 5-week-old L*er* WT, *acd11-1 NahG*, *laz5-D1 acd11-1 NahG*, and *laz5-D2 acd11-1 NahG* after treatment with 100 µM BTH. Means ± s.d. were calculated from 4 discs per treatment with 4 replicates in an experiment. **D**, L*er* WT, *acd11-1* and *laz5-D1 acd11-1* plants 3 weeks after germination.

Transcriptome analysis of genes normally induced in *acd11-1 NahG* after BTH treatment showed that one of the most affected genes in *laz2-1* was *At5g44870*, annotated as an NB-LRR *R* gene (Fig. 3A). This agrees with data from a previous study showing that *At5g44870* is severely down-regulated in *ccr1-1* (*sdg8*) leaf tissues [22]. A number of *acd11* suppressors found in the same screen as *laz2* were dominant. One of these, *laz5 Dominant 1* (*laz5-D1*), was mapped to a region close to this *R* gene (Fig. S5). Sequencing of *At5g44870* in *laz5-D1* revealed a G to A transition at the splice donor site (+1 position) of intron 4 likely resulting in deletion of exon 5 (Fig. 3B). To confirm that this mutation resulted in suppression of *acd11*, two allelic dominant suppressors, *laz5-D2* and *laz5-D3*, were sequenced: both had lesions in *At5g44870* (below), hereafter referred to as *LAZ5*.

*LAZ5* encodes a TIR-class NB-LRR of unknown pathogen specificity with sequence similarity to RPS4 (Fig. S6), an R protein conferring resistance to *Pseudomonas syringae* expressing the effector *AvrRPS4* [30]. The DEB-induced *laz5-D2* mutation is a T to A transversion changing isoleucine 287 to asparagine (I287N). This mutation is within the P-loop motif of the NB domain essential for coordination of bound nucleoside triphosphates [5]. The EMS-induced point mutation in *laz5-D3* (G811E) lies in the LRR domain, which provides pathogen recognition specificity and has been implicated in R protein activation [31]. Accelerated cell death in *acd11-1* was suppressed by *laz5-D1* and *laz5-D2* (Fig. 3C), and *laz5-D* alleles suppressed *acd11* cell death irrespective of BTH induction or the presence of *NahG* (Fig. 3D). Furthermore, over-expression of *laz5-D2* or *laz5-D3* (*35S:laz5-D2 or 3*) suppressed *acd11* death after induction, confirming that dominant negative mutations in *LAZ5* are responsible for suppression of the *acd11*-dependent autoimmune response (Fig. S7).

**Figure 4:**
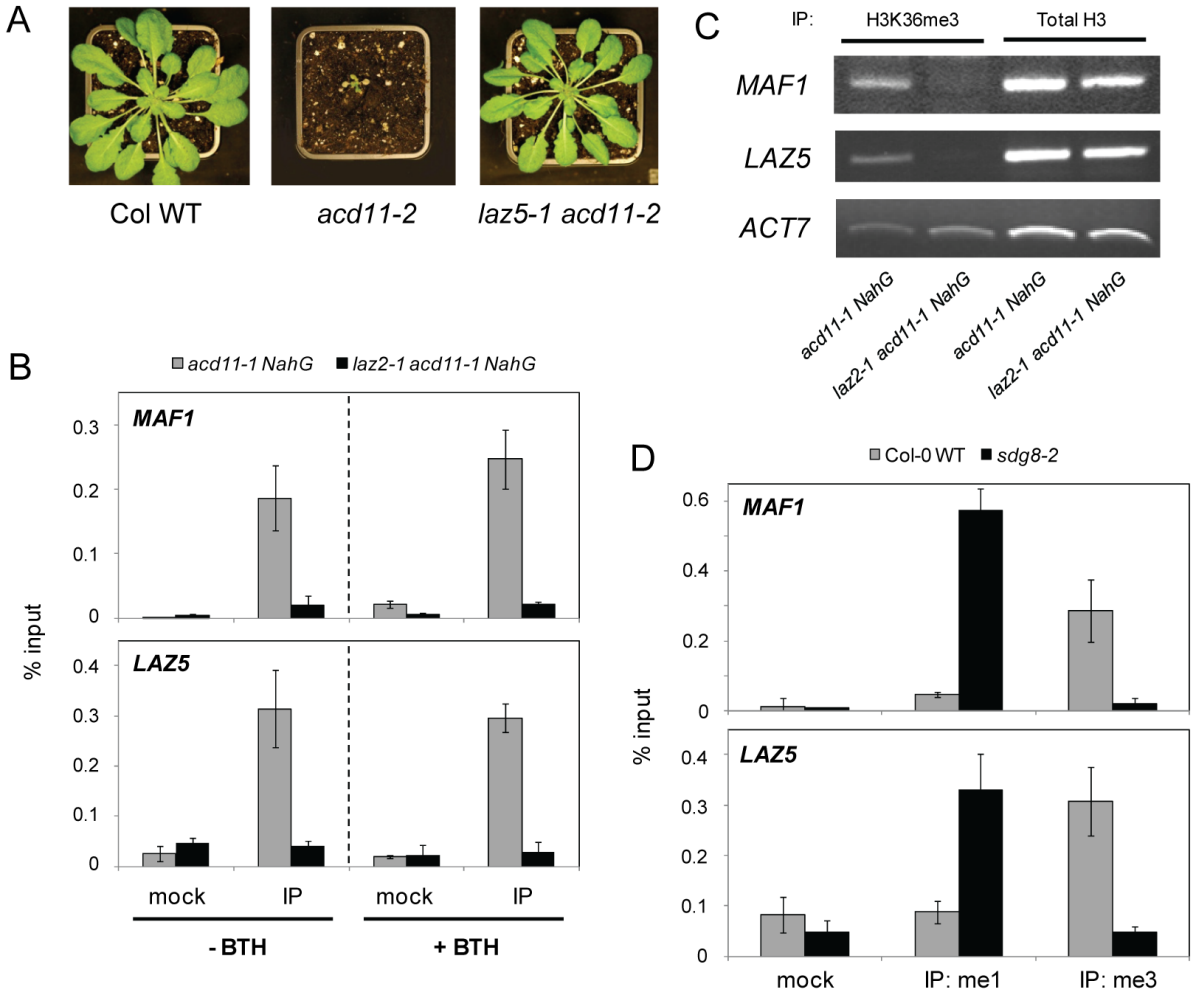
SDG8 regulates LAZ5 and modifies chromatin at the *LAZ5* locus. **A**, Representative 3-week-old Col-0 WT, *acd11-2* (in Col-0) and *laz5-1 acd11-2* plants. **B**, ChIP analysis of *MAF1* and *LAZ5* with 1 µg anti-H3K36-me3 antibody (IP) or no Ab (mock) expressed as proportion of input material in eluates after IP with appropriate Ab (% input), mean ± s.d. (n = 3). Tissue was from 3-week-old *acd11-1 NahG* and *laz2-1 acd11-1 NahG* seedlings (L*er* background) before and 72 h after treatment with 100 µM BTH. The experiment was repeated twice with similar results. **C**, Total H3 levels at *MAF1*, *LAZ5* and *ACT7* chromatin, and H3K36me3 levels at *ACT7*, are not affected by *laz2-1* as determined by ChIP analysis with 1 µg anti-H3K36-me3 antibody or 1 µg anti-H3 (total) antibody, presented as EtBR-stained PCR product (34 cycles). **D**, ChIP of *MAF1* and *LAZ5* with 1 µg anti-H3K36-me1 antibody (me1), 1 µg anti-H3K36-me3 antibody (me3), or no Ab (mock) expressed as % input, mean ± s.d. (n = 3). Tissue was from 3-week-old homozygous *sdg8-2* and Col-0 WT seedlings. The experiment was repeated twice with similar results.

Transgenic plants over-expressing *R*-genes can exhibit spontaneous cell death and/or constitutive defense responses [32]. In agreement with these observations and the phenotype associated with deletion of ACD11, over-expression of wild-type *LAZ5* (*35S:LAZ5*) in the Col-0 background resulted in 30 out of 38 transgenic plants exhibiting *acd11*-like cell death which did not survive to set seed (Fig. S8). Since *LAZ5* transcription is likely dependent on SDG8 HKMT activity, and the suppression of *acd11* by *laz2*/*sdg8* is recessive, we predicted that a loss-of-function mutation in *LAZ5* would suppress *acd11* in a recessive manner. As expected, a null T-DNA insertion mutant of *At5g44870* (SALK_087262; here termed *laz5-1*) suppressed *acd11-2* cell death recessively in plants without *NahG* (Fig. 4A). A second T-DNA insertion mutant allele of *LAZ5* (SAIL_874-D10) also suppressed cell death in *acd11-2* (data not shown). Expression of *LAZ5* was assayed by real-time PCR in wild-type, *laz5-1*, and *sdg8-2* plants 24 hours after syringe inoculation with the virulent bacterial pathogen *Pseudomonas syringae tomato* (*P.s.t.*) DC3000 or with 10 mM MgCl_2_ (mock control). While pathogen treatment induced *LAZ5* expression in wild type, transcript levels in *sdg8-2* were comparable to that in the *laz5-1* null mutant (Fig. S9A). This confirms the microarray expression data shown in Fig. 3A. The apparent lack of *LAZ5* expression in *sdg8-2* was seen in several independent experiments with plants at different stages and/or treated with other pathogen strains (data not shown). Moreover, *ACD11* expression was unaffected in *laz5-1* and *sdg8-2* (Fig. S9B), and transcript accumulation of several TIR-NB-LRR-encoding genes homologous to *LAZ5* was seemingly unaffected in 3-week old *sdg8-2* plants compared to wild-type control with the possible exception of *At5g45230* (Fig. S10).

An important question is whether LAZ5 is the relevant target of SDG8 required for *acd11* cell death. To help answer this question, we transformed *laz2-1 acd11-1 NahG* plants with a genomic construct of *LAZ5* under control of a constitutive promoter and monitored cell death by ion leakage after BTH treatment compared to relevant controls (Fig. S11). *LAZ5* over-expression restored cell death in leaf discs between 3 and 8 days after induction, indicating that lack of *LAZ5* expression in *sdg8* is a major cause of the suppression of *acd11* cell death. However, it cannot be excluded that other targets of SDG8 histone methyltransferase activity also contribute to BTH-induced cell death in *acd11*.

### SDG8 directly modifies chromatin at the *LAZ5* locus

To test whether *laz2* directly affects histone methylation at the *LAZ5* locus, chromatin immunoprecipiation (ChIP) was conducted using antibodies against specifically modified histones. In *laz2-1 acd11-1 NahG*, trimethylated (me3) H3K36 levels were reduced in chromatin associated with the 5′ coding regions of *MAF1* (control) and *LAZ5*, when compared to the *acd11-1 NahG* control (Fig. 4B). Enrichment of H3K36me3 in *LAZ5* chromatin was not influenced by BTH treatment or *acd11* homozygosity (Fig. S12A). This suggests that activation of cell death in *acd11* does not result in hyper-trimethylation at H3K36, but rather that this histone modification is required for proper *LAZ5* expression. There was no effect of genotype on levels of total H3 (Fig. 4C). H3K36me3 is not a general mark for genes up-regulated in *acd11*, such as *FMO1* [18], since we found no enrichment at *FMO1* chromatin 72 h after BTH induction (Fig. S12B, C). Moreover, absence of LAZ2/SDG8 had no effect on H3K36me3 levels at the constitutively expressed *ACTIN* locus (Fig. 4C) or the *MAP KINASE KINASE 4* (*MKK4*) locus (Fig. S12D).

To elucidate H3K36 methylation status irrespective of *acd11* and *NahG*, we also conducted ChIP assays on *sdg8-2* single mutant and Col-0 wild-type seedlings. It was previously shown that loss of SDG8 resulted in both a decrease in global H3K36me3 levels and a coincident increase in global monomethylated (me1) H3K36, a mark associated with transcriptional repression in *Arabidopsis* [25]. In wild-type plants, *MAF1* and *LAZ5* chromatin was enriched for H3K36me3, whereas the level of H3K36me3 was diminished in *sdg8-2* (Fig. 4D). Conversely, H3K36me1 levels at these loci were higher in *sdg8-2* and reduced in wild type. Treatment of seedlings for 3 hours with an HR-inducing bacterial pathogen had no effect on the methylation status of H3K36 (data not shown). Also, H3 trimethylation of *LAZ5* chromatin at other lysine residues (K4, K9, K27), was not affected by loss of SDG8 (Fig. S12E).

### SDG8 is required for pathogen resistance in Arabidopsis

**Figure 5:**
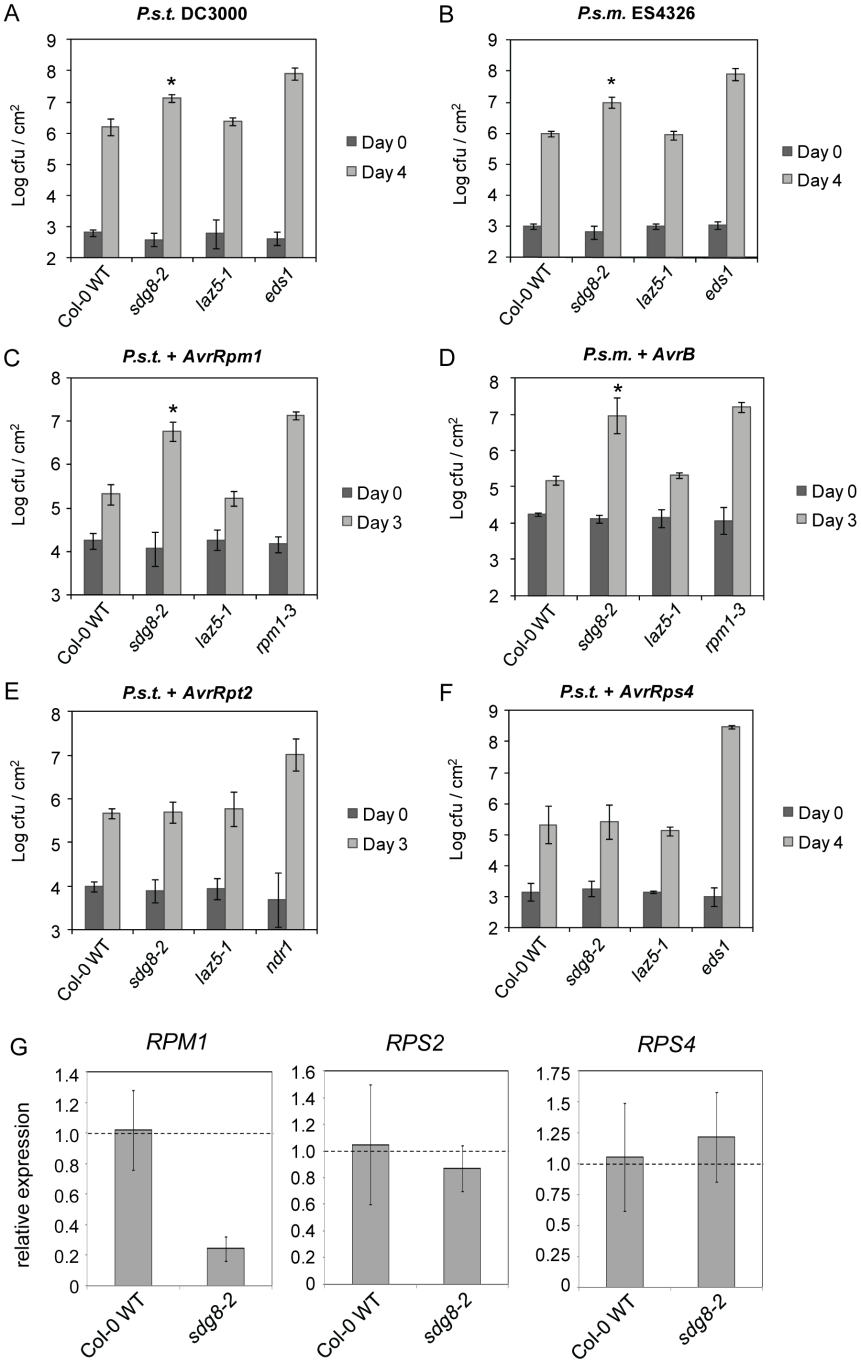
SDG8 is required for plant innate immunity. **A**, Growth of virulent *P.s.t.* DC3000 in Col-0 WT, *sdg8-2*, *laz5-1* and *eds1* 0 and 4 days after infiltration with bacteria at OD_600_ = 0.0001. **B**, Growth of virulent *P.s.m.* ES4326 in 3-week old Col-0 WT, *sdg8-2*, *laz5-1* and *eds1* plants 0 and 4 days after infiltration with bacteria at OD_600_ = 0.0001. **C**, Growth of avirulent *P.s.t.* DC3000 expressing *AvrRpm1* in Col-0 WT, *sdg8-2*, *laz5-1* and *rpm1-3* 0 and 3 days after infiltration with bacteria at OD_600_ = 0.001. **D**, Growth of avirulent *P.s.m.* ES4326 expressing *AvrB* in Col-0 WT, *sdg8-2*, *laz5-1* and *rpm1-3* plants 0 and 3 days after infiltration with bacteria at OD_600_ = 0.001. **E**, Growth of avirulent *P.s.t.* DC3000 expressing *AvrRpt2* in Col-0 WT, *sdg8-2*, *laz5-1* and *ndr1* 0 and 3 days after infiltration with bacteria at OD_600_ = 0.001. **F**, Growth of avirulent *P.s.t.* DC3000 expressing *AvrRps4* in Col-0 WT, *sdg8-2*, *laz5-1* and *eds1* plants 0 and 4 days after infiltration with bacteria at OD_600_ = 0.00005. Log-transformed values are means ± s.d. (n = 6). Asterisks indicate statistical significance (p<0.0001) determined by unpaired Student's t-test. The experiments were repeated twice with similar results. cfu = colony forming units. **G**, Transcript accumulation of *RPM1*, *RPS2*, and *RPS4* in 3-week-old Col-0 wild-type and *sdg8-2* plants, as determined by qRT-PCR. Data is normalized to *ACTIN1* (*ACT1*) and presented as relative expression compared to Col-0 = 1.0, mean ± s.d. (n = 3).

To determine whether SDG8 and/or LAZ5 are required for basal resistance to virulent pathogens, leaves of 4-week old *sdg8-2*, *laz5-1*, wild-type and an allele of *enhanced disease susceptibility 1* (*eds1-2* introgressed into Col-0) mutants were syringe-inoculated with *P.s.t.* DC3000 and growth was assayed after 4 days. Bacteria grew to ∼9-fold higher titers in *sdg8-2* than in wild-type or *laz5-1*, while titers in *eds1* were yet another order of magnitude higher (Fig. 5A). Growth of another strain of bacterial pathogen, *Pseudomonas syringae maculicola* (*P.s.m.*) ES4326, was tested on *sdg8-2*, *laz5-1*, wild-type and *eds1* with similar results (Fig. 5B). We did not observe elevated bacterial growth in *sdg8-2* when we used *P.s.t.* DC3000 *HrcC*- (Fig. S13A), a non-pathogenic mutant defective in delivery of effectors to host cells [33]. These data indicate that SDG8, but not LAZ5, is required for full resistance to virulent pathogens. Furthermore, we found that SDG8 is involved in resistance to avirulent pathogens mediated by other R proteins, for example RPM1. Plants were syringe-inoculated with *P.s.t.* DC3000 expressing HR-inducing *AvrRpm1*, *AvrRpt2*, *AvrRps4* or *AvrPphB* and growth was assayed after 3 or 4 days. Bacterial titers were ∼15-fold higher in *sdg8-2* than in wild-type or *laz5-1* for *P.s.t.* expressing *AvrRpm1* (Fig. 5C). This suggested that *RPM1*-mediated resistance is defective in *sdg8-2*. To confirm this, growth of *P.s.m.* ES4326 expressing *AvrB* was assessed after 3 days: *AvrB* is also recognized by RPM1, and resistance to this avirulent pathogen was affected in *sdg8-2* to a similar level as *P.s.t.* with *AvrRpm1* (Fig. 5D). In both cases, bacterial titers were comparable to the *rpm1-3* null mutant [34]. Defects in SDG8 had a consistent, yet statistically insignificant effect on growth of *P.s.t.* DC3000 expressing *AvrPphB*, (Fig. S13B) resistance to which is dependent on the *R* gene *RPS5* [35]. In addition, *sdg8-2* did not affect RPS2- or RPS4-mediated resistance to *AvrRpt2* [36], [37] (Fig. 5E) and *AvrRps4* [30] (Fig. 5F). Corroborating the pathogen growth assay, transcript levels of *RPM1* and *RPS5* were low or absent in 4-week old *sdg8-2* compared to wild-type, whereas expression of *RPS2* and *RPS4* in *sdg8-2* was similar to that in wild-type (Fig. 5G and S13C). Defects in *LAZ5* did not have a detectable effect on transcript accumulation of *RPM1*, *RPS5*, *RPS2* or *RPS4* (data not shown). As with *LAZ5*, we conducted ChIP assays at the *RPM1* locus in untreated seedling tissue from *laz2-1 acd11-1 NahG* versus *acd11-1 NahG* (in L*er*) and *sdg8-2* versus wild-type (in Col-0). We observed lower H3K36me3 and higher H3K36me1 levels at *RPM1* chromatin in the absence of functional *LAZ2*/*SDG8*, indicating that *RPM1* is an example of another *R* gene that is regulated by histone methylation (Fig. S14). These results indicate that SDG8 targets a subset of *R* genes and other genes involved in more general aspects of basal defense.

## Discussion

Chromatin remodeling has emerged as a complex regulator of transcription and an epigenetic mechanism to maintain lasting changes in gene activity states. Dynamic post-translational modifications of various residues of histones tails, including methylation, phosphorylation, acetylation, and ubiquitination, play important roles in both promoting and repressing gene expression by recruiting histone binding proteins and chromatin remodeling enzymes [38]. The combinatorial nature of histone modifications results in a complex “histone code” that adds an important level of control to fine-tune gene-specific responses to broader transcriptional inputs [39]. Changes in chromatin state may therefore modulate gene expression in a context-dependent manner to maintain a flexible response to pathogen attack. In plants, this process has been proposed as a mechanism for priming SA-responsive loci during systemic acquired resistance to pathogens [40].

So far, relatively few studies directly associate epigenetic processes related to chromatin modification to plant innate immunity and/or PCD. Defects in *HISTONE DEACETYLASE 19* (*HDAC19*) and *HISTONE MONOUBIQUITINATION 1* (*HUB1*) increase susceptibility to necrotrophic fungal pathogens in *Arabidopsis* [41], [42]. Furthermore, defects in genes involved in histone variant replacement, and the variant H2A.Z itself, result in increased resistance to virulent bacterial pathogens, some spontaneous cell death, and up-regulation of defense genes [43]. More commonly, the “memory” of chromatin remodeling activity is observed as increased levels of open chromatin marks (H3Ac, H3K4me2, etc) at the promoters of many SA-responsive genes, such as *PATHOGENESIS-RELATED 1* (*PR-1*) and WRKY TFs [40], [44], [45]. The clearest example of immune response at the level of chromatin comes from Alvarez-Venegas and colleagues, who showed that the HKMT ARABIDOPSIS TRITHORAX 1 (ATX1, also known as SDG27) controls expression of WRKY70, a TF involved in pathogen response [46]. ATX1-dependent H3K4me3 signatures at the promoter of *WRKY70* correlated with *WRKY70* transcriptional up-regulation. Intriguingly, although ATX1 regulates expression of a large set of genes, a high proportion of immunity-related genes exhibited reduced expression in the knockout mutant, including various *TIR-NB-LRR R* genes [47]. Numerous examples exist of microbes and viruses manipulating host chromatin remodeling machinery or histones directly in animals [48], [49]. Strikingly, toxins from unrelated bacterial pathogens of animals have evolved to modify host histones, reducing transcriptional activity of key immunity genes [50]. The only clear instance of related phenomena identified among plant pathogens is the case of the Crown Gall disease-causing bacterium *Agrobacterium tumefaciens* which selectively modulates the expression of host variant histone genes to allow genomic integration of its T-DNA [51], [52].

There is conflicting data on whether loss of *sdg8* influences H3K4 methylation, H3K36 methylation, or both [22], [23], [25], [28]. We detected a dramatic effect of *laz2*/*sdg8* on H3K36 methylation status of chromatin at various loci and no difference in H3K4me3 levels at LAZ5, although the H3K4 methylation status of chromatin at other loci in *laz2* backgrounds remains to be investigated. In addition, our data suggest that monomethylation of H3K36 at *MAF1* and *LAZ5* chromatin relies on HKMTs other than SDG8. One of these, SDG26, was previously shown to act antagonistically to SDG8 by repressing FLC expression, although global H3K36me1 levels were unaffected in the *sdg26* mutant [25]. The significance of H3K36me1 enrichment in *sdg8-2* remains unknown. One hypothesis is that H3K36 methylation proceeds in a stepwise fashion, with the accumulation of H3K36me1 (due to activity of an unknown HKMT) being a consequence of a block in further di- and trimethylation at this residue normally mediated by SDG8. Alternatively, monomethylation of H3K36 may represent a transcriptionally repressive mark that accumulates only in the absence of di- and trimethylation due to disruption of the balance between antagonistic chromatin modifiers. For example, the SET-domain containing *Arabidopsis* proteins TRITHORAX-RELATED PROTEIN 5 (ATXR5, also known as SDG15) and ATXR6/SDG34 are H3K27-specific monomethyltransferases essential for transcriptional repression in heterochromatin [53]. Further studies should examine if other predicted H3K36-specific HKMTs, namely SDG4, SDG7, SDG24 and SDG26, have any role in H3K36 monomethylation, trimethylation and/or antagonistic control of expression of *LAZ5* and other genes with roles in immunity or are required for cell death in *acd11*. Moreover, further work is required to determine the mechanisms by which SDG8-dependent changes in H3 methylation regulates the expression of specific genes.

A clue to the function of LAZ5 activation comes from the isolation in our screen of dominant alleles. This indicates that the mutant form (*laz5-D*) of the R protein likely interferes with activity of the wild-type copy since plants heterozygous for the *laz5* null mutation do not suppress *acd11*, indicating haplosufficiency of *LAZ5*. Dominant negative activity has been described for mutations in the *R* gene *N* from tobacco, and indeed for a point mutation (G216E) in the P-loop motif of N [54]. N was later found to oligomerize in the presence of a Tobacco mosaic virus elicitor, likely through interaction of TIR domains [55]. This oligomerization was an early event in pathogen perception and was independent of mutations that have an effect on HR induction. Therefore, it is possible that *laz5-D* mutants form inactive oligomers with wild-type LAZ5 and/or accessory proteins. An example of this scenario from animal innate immunity comes from NOD2, an NLR involved in recognition of bacterial cell wall components: an endogenously truncated form, NOD2-S, interacts with full-length NOD2 to potentiate signaling [56]. In plants, there are examples of truncated R proteins, generated by alternative splicing, playing a key role in signaling [57], [58]. At present, it is an open question whether LAZ5 oligomerizes and how this relates to cell death activation. It should be noted that, while all the *laz5* alleles isolated thus far in the *acd11* suppressor screen were dominant negative, only 43 of the ∼200 unknown recessive mutants were placed into complementation groups, and even fewer were mapped. Therefore, a recessive *laz5* knockout allele may exist among our unmapped suppressors.

In this study we have identified the chromatin modifying enzyme SDG8, and its specific target LAZ5, as regulators of autoimmune cell death in *acd11*. Furthermore, *sdg8* mutants exhibit enhanced susceptibility to virulent and avirulent pathogens, whereas *laz5* mutants do not, suggesting that other targets of SDG8 are important for general resistance. We also show that transcription of a subset of *R* genes, including *LAZ5* and *RPM1*, is likely to be directly or indirectly dependent on LAZ2 activity. One scenario that may account for the enhanced susceptibility of *sdg8* mutants to virulent pathogens could be the consequence of SDG8 action on multiple NB-LRR loci. If the suite of effectors delivered by *Pseudomonas* triggers a weak *R* gene response, in *sdg8* a subset of these do not accumulate and thus are no longer available to signal for defense against the invading pathogen. Intriguingly, *SDG8* is not expressed until 8 days after germination [28], a stage preceding the initiation of cell death in *acd11*. SDG8 may therefore developmentally regulate targets such as *LAZ5*, and may exemplify a key difference in the programmed defenses required during seed maturation and the inducible defenses used during plant growth.

Lesion mimic mutants such as *acd11* are useful tools in the genetic dissection of innate immunity in plants [10]. Whereas several of these mutants have putative roles in ceramide signaling or synthesis [59], [60] or auto-activate R proteins [11], the majority of lesion mimic mutants represent proteins with no straightforward connection to PCD. Milder autoimmunity, associated with constitutive activation of defense responses and dwarf morphology without coincident HR, can similarly be the result of point mutations in immune receptors (Zhang et al., 2003), or deletion of signaling intermediates such as MAP kinases [61]. Knockout mutants that eliminate host guardees mimic the effects of pathogen effectors, and have been found to exhibit *R*-gene-dependent lethality [62]. Therefore, it is possible that many lesion mimic/autoimmune mutants may correspond to gene functions that are guarded by NB-LRRs. If so, the diverse functions of these genes may be “red herrings” not directly related to PCD but only implicated in this process due to their targeting by pathogen effectors. Such may be the case for *acd11*, although we have been unable to detect any interaction between full-length or truncated LAZ5 and ACD11 in yeast or *in planta* (data not shown). Previously, we reported the identification of ACD11-interacting proteins [63], which we are testing for interaction with LAZ5. Two predictions about wild-type products of autoimmune mutants emerge from this model. First, suppressor screens should identify *R* genes. Second, pathogen effectors should target them either directly or indirectly via interacting partners or products of their activities. We currently have no evidence that ACD11 is targeted by pathogen effectors, or that ACD11 contributes to disease resistance in the absence of LAZ5. While future work may strengthen this hypothesis, an alternative model is that ACD11 is involved in negatively regulating SA-dependent expression of *LAZ5* (or a subset of *R* genes) perhaps via some lipid signal.

## Materials and Methods

### Plant material and growth conditions

*Arabidopsis* plants were grown on soil or MS-agar plates at 21°C with an 8 h or 12 h photoperiod. *sdg8-2* (*SALK_026642*) and *laz5-1* (*SALK_087262*) T-DNA insertion lines, both previously described as null mutants [23], [64], were generated by SIGnAL [65] and obtained from the Nottingham *Arabidopsis* Stock Centre (NASC; Nottingham, UK). Homozygous genotyping primers were 5′-TAAAGAGGGTCTGCATCATGG-3′ with 5′-CACTGTCCAGTAAAAGCTGGC-3′ for *sdg8-2* and 5′-TATGTTTTTCCCAGATGCCAG-3′ with 5′-ATCATGCATCTCAACTCGACC-3′ for *laz5-1*. Sequences of primers used to detect *acd11-1*, *acd11-2*, and *NahG* are available upon request.

### Suppressor screen

Three lots of 920–950 mg L*er acd11-1 NahG* seeds were incubated for 4 hr in either 0.74% (w/v) EMS (Sigma-Aldrich, St Louis, MO, USA) prepared in 0.1M sodium phosphate buffer, pH 5, with 5% DMSO, or 10 mM DEB (Sigma-Aldrich) in water, followed by rinsing. γ-irradiation of 300 mg *acd11-1 NahG* seeds was performed at the Risø Reference Laboratory (Denmark) with 500 Gy from a Cobalt-80 source. M_1_ plants were grown in families of 125 individuals, 3500 M_2_ plants per family were screened for BTH-resistant suppressors. ∼3 million M_2_ plants from 845 M_1_ pools or ∼100.000 M_1_ plants were scored. Putative mutants were genotyped to be homozygous for *acd11-1* by PCR.

### Ion leakage assay

Conductivity assays were conducted essentially as previously described [66].

### Microarray hybridization

Total RNA was isolated from three independent biological replicates of relevant genotypes at 0 and 72 hr after BTH treatment. RNA was labeled and amplified according to the MessageAmp Biotin-enhanced kit (Ambion) protocol and hybridized to 51 ATH1 GeneChips after Affymetrix protocols.

### Chromatin immunoprecipitation and real-time PCR

ChIP antibodies purchased from Abcam (Cambridge, UK) included anti-H3 (ab1791), anti-H3K36me1 (ab9048), anti-H3K36me3 (ab9050) and anti-H3K27me3 (ab6002). ChIP antibodies against H3K4me3 (pAb-056-050) and H3K9me3 (pAb-003-050) were purchased from Diagenode (Liège, Belgium). Quantitative PCR primers for ChIP analysis were *LAZ5*: 5′-GAGTCGTGGCAAGTGTTCATC-3′ with 5′- GAAGATGGACAGTGCGATTTC-3′; *FMO1*: 5′-CTCAGATGGCTTCTAACTATG-3′ with 5′-CTATTATTGGGCCATGGAAAG-3′; *MAF1*: 5′-CCCTTATCGGAGATTTGAAGC-3′ with 5′-GGAGGATTCACAGAGAATCG-3′; *ACTIN*: 5′-GGAAACATCGTTCTCAGTGG-3′ with 5′-ACCAGATAAGACAAGACACAC-3′. ChIP was performed essentially as described [67], using 1µg of each antibody. Real-time PCR to quantify the immunoprecipitated DNA was performed using Brilliant II SYBR Green qPCR kit (Stratagene), and reactions were run on an iCycler IQ (Bio-Rad, Hercules, CA, USA). In all cases, ChIP values were calculated using the Delta-Delta-Ct (ddCt) algorithm to determine relative gene expression utilizing the ‘percent input method’. Briefly, signals obtained from the ChIP were divided by signals obtained from an input sample representing the amount of chromatin used in the ChIP. The ‘% input’ value shows what proportion of this starting material is found in the eluate after IP with appropriate Ab.

For expression analyses, RNA was extracted from relevant genotypes using the Qiagen RNeasy RNA extraction kit followed by DNase treatment as per the manufacturer's instructions. Equal amounts of RNA were subjected to one-step real-time PCR using the same kit as described for ChIP except with reverse transcriptase included. For all sample/primer combinations, a control without reverse transcriptase was included to exclude genomic DNA contamination.

### Cloning and generation of transgenic plants

3.9-kb fragments of *laz5-D* alleles were amplified from genomic DNA (*laz5-D1 acd11-2 NahG*, *laz5-D2 acd11-2 NahG*, *laz5-D3 acd11-2 NahG*) and cloned into modified pCAMBIA-3300 as described [68], using a uracil-excision based cloning technique (USER, New England Biolabs). Cloning primers were 5′-ggcttaaUATGGCAGCATCTTCCGAAATAC-3′ and 5′-ggtttaaUTTACAATAAACCCAAGTATAATTTAG-3′. A 3.9-kb fragment of *LAZ5* was amplified from genomic DNA (wild type L*er*), cloned into pENTR/D-TOPO (Invitrogen) and transferred to Gateway-compatible constitutive expression vectors pGWB502Ω or pGWB521 [69] by LR recombination reaction (Invitrogen). Cloning primers used were 5′-CACCATGGCAGCATCTTCCGAAATAC-3′ and 5′-TTACAATAAACCCAAGTATAATTTAG-3′. The final constructs were verified by sequencing, electroporated into *Agrobacterium tumefaciens* strain GV3101 and used to transform *acd11-1 NahG* or wild type plants by floral dip method [70]. Transgenic plants were selected on soil with glufosinate (*35S:laz5-D* alleles) or on MS-agar plate with (20mg/L) hygromycin B followed by transplanting to soil (*35S:LAZ5*).

### Accession numbers

*At2g34690* (ACD11): NP_181016. *At1g77300* (LAZ2/SDG8): NP_177854. *At5g44870* (LAZ5): NP_199300. *At1g77080* (*MAF1*): NM_180648. *At5g10140* (*FLC*): NM_121052. *At1g19250* (FMO1) NP_173359. *At5g09810* (ACTIN): NP_196543. *At1g06820* (CRTISO): NP_172167. *At3g48090* (*EDS1*) NM_114678. *At3g20600* (NDR1): NP_188696. *At3g07040* (RPM1): NP_187360. *At4g26090* (RPS2): NP_194339. *At5g45250* (RPS4): NP_199338. At1g12220 (RPS5): NP_172686. *At5g17880* (CSA1): NP_197290. *At4g36150*: NP_195338. *At5g45200*: NP_199333. *At5g45230*: NP_199336.

## Supporting Information

Figure S1 *laz2* alleles and *sdg8* share morphological phenotypes, such as early flowering. **A**, 16-day-old L*er acd11-1 NahG* plants homozygous for 3 different *laz2* alleles. **B**, 21-day-old Col-0 WT plants homozygous for *sdg8-2*.
(3.77 MB TIF)

Figure S2 Transcriptome analysis of *laz2-1* suppression of the BTH-induced response in *acd11-1* **A**, The effect of *laz2-1* on 355 significantly over-expressed genes among the top 500 differentially expressed genes in response to BTH treatment in *acd11-1 NahG* plants. **B**, Scatterplot of global expression fold change comparison between *acd11-1 NahG* versus *NahG* (y-axis) and *laz2-1 acd11-1 NahG* versus *acd11-1 NahG* (x-axis) 72 h after BTH induction.
(0.41 MB TIF)

Figure S3 Ecotype-specific markers used to map the *LAZ2* locus to ∼120 kb on the bottom of chromosome 1. Left is centromeric, right is telomeric. Relative positions of markers are indicated, as are numbers of recombinants remaining at each marker position. Figure shows a rough (∼1 megabase) and fine (∼150-kb) map of the *laz2-1* locus and detail of genomic region between final recombinants, with associated genes and BAC clones. A star marks the *LAZ2* gene with the defect determined by sequencing.
(0.19 MB TIF)

Figure S4 Expression of (**A**) *CRTISO* (*At1g06820*) and (**B**) *MAF1* (*At1g77070*) in L*er* WT, *NahG*, *acd11-1 NahG* and *laz2-1 acd11-1 NahG* before and 72 h after treatment with 100 µM BTH relative to WT at time point 0 (log2 scale).
(0.12 MB TIF)

Figure S5 Ecotype-specific markers were used to map the *LAZ5* locus to ∼80 kb on the bottom of chromosome 5. Left is centromeric, right is telomeric. Relative positions of mapping markers and numbers of recombinants are indicated. Figure shows a map of the *laz5-D1* locus and the genomic region between final recombinants, with associated genes. Asterisk marks the *LAZ5* gene with the defect determined by sequencing.
(0.24 MB TIF)

Figure S6 Alignment of LAZ5 and the five most similar Arabidopsis TIR-NB-LRR R proteins, as determined by The Functional and Comparative Genomics of Disease Resistance Gene Homologs Project (http://niblrrs.ucdavis.edu/TN_TNL_phylogeny.html). Sequences include RPS4 (At5g45250), CSA1 (At5g17880), At4g36150, At5g45200, and At5g45230. Mutated residues in *laz5-D2* and *laz5-D3* are highlighted. Asterisks indicate amino acids predicted to be absent due to the splice site mutation in *laz5-D1*.
(1.35 MB TIF)

Figure S7 Over-expression of dominant negative *laz5-D* alleles suppresses *acd11*. Figure shows *acd11-2 NahG* (in Col-0) control and representative transgenic lines of *acd11-2 NahG* stably transformed with (**A**) *35S:laz5-D2* or (**B**) *35S:laz5-D3*, 10 d after treatment with 100 µM BTH.
(1.70 MB TIF)

Figure S8 Over-expression of the wild-type *LAZ5 R* gene results in cell death. Figure shows Col-0 wild-type control and two representative transgenic lines of Col-0 stably transformed with a construct over-expressing genomic *LAZ5* (*35S:LAZ5*).
(2.43 MB TIF)

Figure S9 Expression of (**A**) *LAZ5* and (**B**) *ACD11* in 3-week-old Col-0 wild-type, *laz5-1* and *sdg8-2* mutant plants 24 h after infiltration with *P.s.t.* DC3000 at OD_600_ = 0.001 or 10mM MgCl_2_ mock control, as determined by qRT-PCR. Data is normalized to *ACTIN1* (*ACT1*) and presented as relative expression (fold) compared to Col-0 mock = 1.0 (dashed line), mean ± s.d. (n = 3).
(0.15 MB TIF)

Figure S10 Transcript accumulation of *LAZ5* homologs in 3-week-old Col-0 wild-type and *sdg8-2* plants, as determined by qRT-PCR. Data is normalized to *ACT1* and presented as relative expression compared to Col-0, mean ± s.d. (n = 3).
(0.23 MB TIF)

Figure S11 Ion leakage cell death assay of leaf discs from 3-week-old WT, *laz2-1 acd11-1 NahG*, *acd11-1 NahG* and *laz2-1 acd11-1 NahG* over-expressing *LAZ5* plants after BTH treatment. The former were selected segregating T_2_ plants from a transgenic line of genomic *LAZ5* in expression vector pGWB521, and confirmed by RT-PCR. Data is presented as fold change in conductivity (µS cm^−1^) relative to initial value at Day 3. Means ± s.d. were calculated from 6 discs per treatment with 4 replicates within an experiment.
(0.13 MB TIF)

Figure S12 **A**, H3K36me3 at *LAZ5* chromatin is independent of *acd11*. ChIP analysis of *LAZ5* with 1 µg anti-H3K36-me3 antibody (IP) or no Ab (mock) expressed as % input. Tissue was from 3-week-old *NahG*, *acd11-1 NahG* and *laz2-1 acd11-1 NahG* seedlings (L*er* background) before and 24 h after treatment with 100 µM BTH. **B**, H3K36me3 is not a general mark for genes up-regulated in *acd11*. Expression of *FMO1* (*At1g19250*) in L*er* WT, *acd11-1 NahG* and *laz2-1 acd11-1 NahG* before and 72 h after treatment with 100 µM BTH relative to wild-type at time point 0 (log2 scale). **C**, ChIP analysis of *FMO1* with 1 µg anti-H3K36-me3 antibody (IP) or no Ab (mock) expressed as % input. Tissue was collected from 3-week-old seedlings. Experiments were repeated twice with similar results. **D**, H3K36me3 levels at the *MKK4* locus is not affected by *laz2-1* as determined by ChIP analysis with 1 µg anti-H3K36-me3 antibody or 1 µg anti-H3 (total) antibody, presented as EtBR-stained PCR product (34 cycles). **E**, Levels of H3K4me3, H3K9me3, H3K27me3 and total H3 at *LAZ5* chromatin are not affected by *sdg8-2* as determined by ChIP analysis with appropriate antibody. In parallel, ChIP samples were used as templates for PCR at the transcriptionally repressed transposon *Ta3* locus for comparison. Data is presented as EtBR-stained PCR product (34 cycles).
(0.32 MB TIF)

Figure S13 **A**, Growth of non-pathogenic *P.s.t.* DC3000 hrcC- mutant in Col-0 WT and *sdg8-2* 0 and 3 days after infiltration with bacteria at OD_600_ = 0.001. **B**, Growth of avirulent *P.s.t.* DC3000 expressing *AvrPphB* in Col-0 WT, *sdg8-2*, *laz5-1* and *ndr1* plants 0 and 4 days after infiltration with bacteria at OD_600_ = 0.001. Log-transformed values are means ± s.d. (n = 6). The experiments were repeated once or twice with similar results. cfu = colony forming units. **C**, Transcript accumulation of *RPS5* in 3-week-old Col-0 wild-type and *sdg8-2* plants, as determined by qRT-PCR. Data is normalized to *ACTIN1* (*ACT1*) and presented as relative expression compared to Col-0 = 1.0, mean ± s.d. (n = 3).
(0.16 MB TIF)

Figure S14 **A**, ChIP analysis of *RPM1* with 1 µg anti-H3K36-me3 antibody (IP) or no Ab (mock) expressed as proportion of input material in the eluate after IP with appropriate Ab (% input), mean ± s.d. (n = 3). Tissue was from 3-week-old *acd11-1 NahG* and *laz2-1 acd11-1 NahG* seedlings (L*er* background) 72 h after treatment with 100 µM BTH. The experiment was repeated twice with similar results. **B**, ChIP of *RPM1* with 1 µg anti-H3K36-me1 antibody (me1), 1 µg anti-H3K36-me3 antibody (me3), or no Ab (mock) expressed as % input, mean ± s.d. (n = 3). Tissue was from 3-week-old homozygous *sdg8-2* and Col-0 WT seedlings. The experiment was repeated with similar results.
(0.12 MB TIF)
