# Correction: Autoimmunity in Arabidopsis *acd11* Is Mediated by Epigenetic Regulation of an Immune Receptor

**DOI:** 10.1371/annotation/c70c3fcc-01df-4f98-aa4f-19697ccf7cfa

**Published:** 2010-10-20

**Authors:** Kristoffer Palma, Stephan Thorgrimsen, Frederikke Gro Malinovsky, Berthe Katrine Fiil, H. Bjørn Nielsen, Peter Brodersen, Daniel Hofius, Morten Petersen, John Mundy

An article cited in the third sentence of the last paragraph in the discussion section is missing from the References. The sentence reads: Milder autoimmunity, associated with constitutive activation of defense responses and dwarf morphology without coincident HR, can similarly be the result of point mutations in immune receptors (Zhang et al., 2003). The reference is: Zhang Y, Goritschnig S, Dong X, Li X (2003) A gain-of-function mutation in a plant disease resistance gene leads to constitutive activation of downstream signal transduction pathways in suppressor of npr1-1, constitutive 1. Plant Cell 15: 2636-2646. 

