# Supplementary figures and images for: Autoimmunity in Arabidopsis acd11 Is Mediated by Epigenetic Regulation of an Immune Receptor
Source: PLoS Pathog. 2010 Oct 7;6(10):e1001137. doi: 10.1371/journal.ppat.1001137 (PMC2951382; doi:10.1371/journal.ppat.1001137)

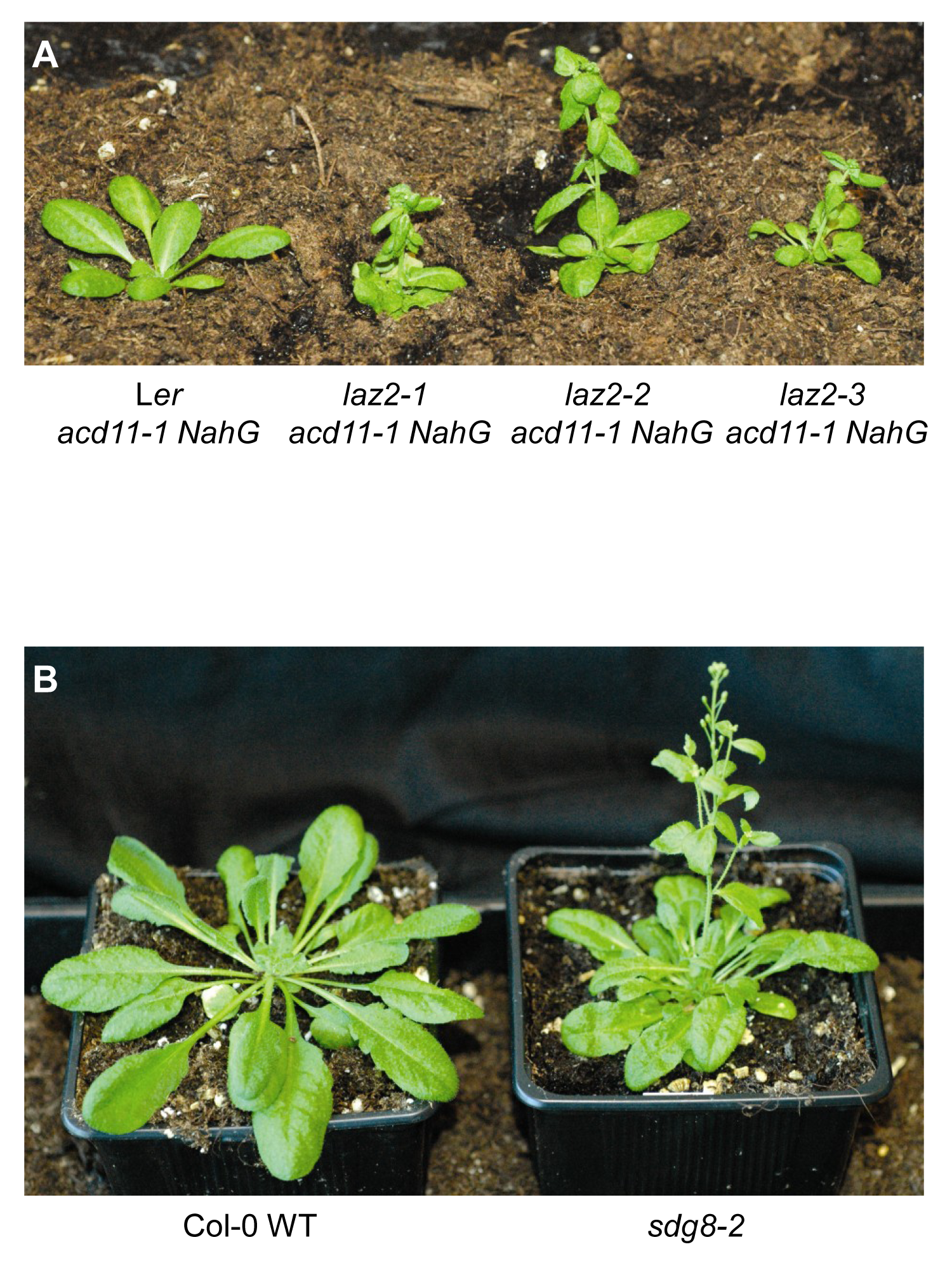

Supplement: Figure S1 — laz2 alleles and sdg8 share morphological phenotypes, such as early flowering. A, 16-day-old Ler acd11-1 NahG plants homozygous for 3 different laz2 alleles. B, 21-day-old Col-0 WT plants homozygous for sdg8-2. (3.77 MB TIF) [file ppat.1001137.s001.tif]

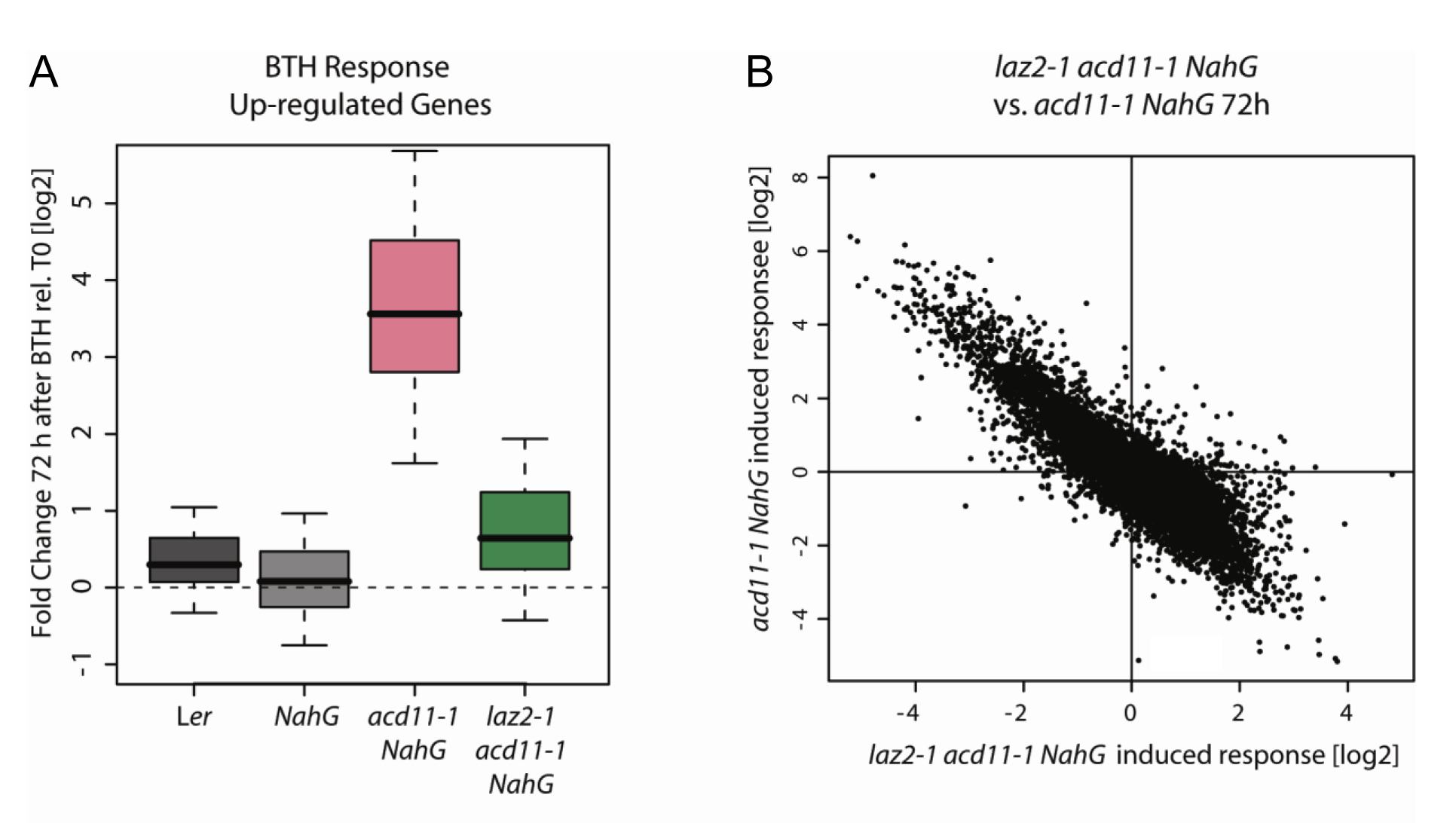

Supplement: Figure S2 — Transcriptome analysis of laz2-1 suppression of the BTH-induced response in acd11-1 A, The effect of laz2-1 on 355 significantly over-expressed genes among the top 500 differentially expressed genes in response to BTH treatment in acd11-1 NahG plants. B, Scatterplot of global expression fold change comparison between acd11-1 NahG versus NahG (y-axis) and laz2-1 acd11-1 NahG versus acd11-1 NahG (x-axis) 72 h after BTH induction. (0.41 MB TIF) [file ppat.1001137.s002.tif]

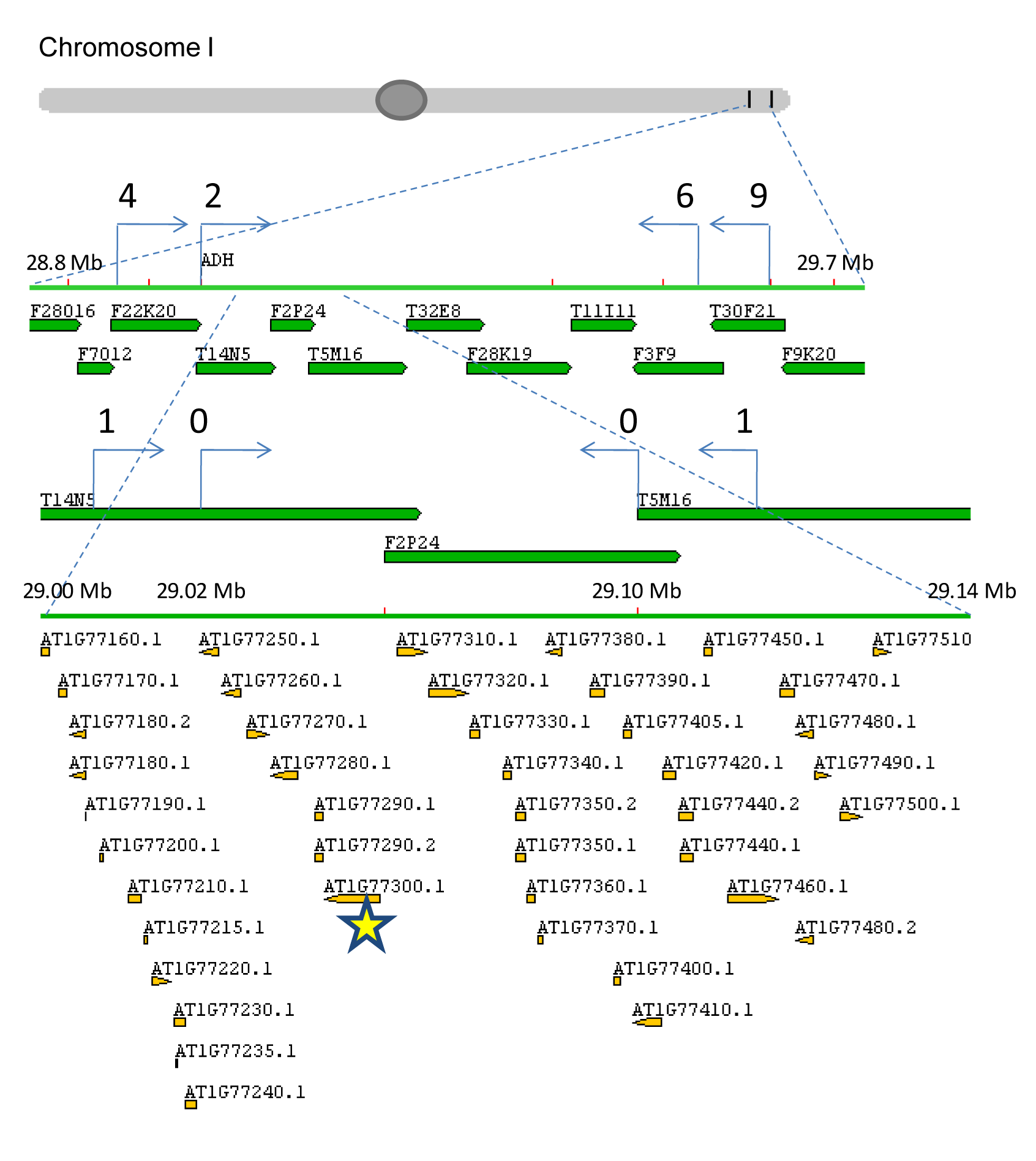

Supplement: Figure S3 — Ecotype-specific markers used to map the LAZ2 locus to ∼120 kb on the bottom of chromosome 1. Left is centromeric, right is telomeric. Relative positions of markers are indicated, as are numbers of recombinants remaining at each marker position. Figure shows a rough (∼1 megabase) and fine (∼150-kb) map of the laz2-1 locus and detail of genomic region between final recombinants, with associated genes and BAC clones. A star marks the LAZ2 gene with the defect determined by sequencing. (0.19 MB TIF) [file ppat.1001137.s003.tif]

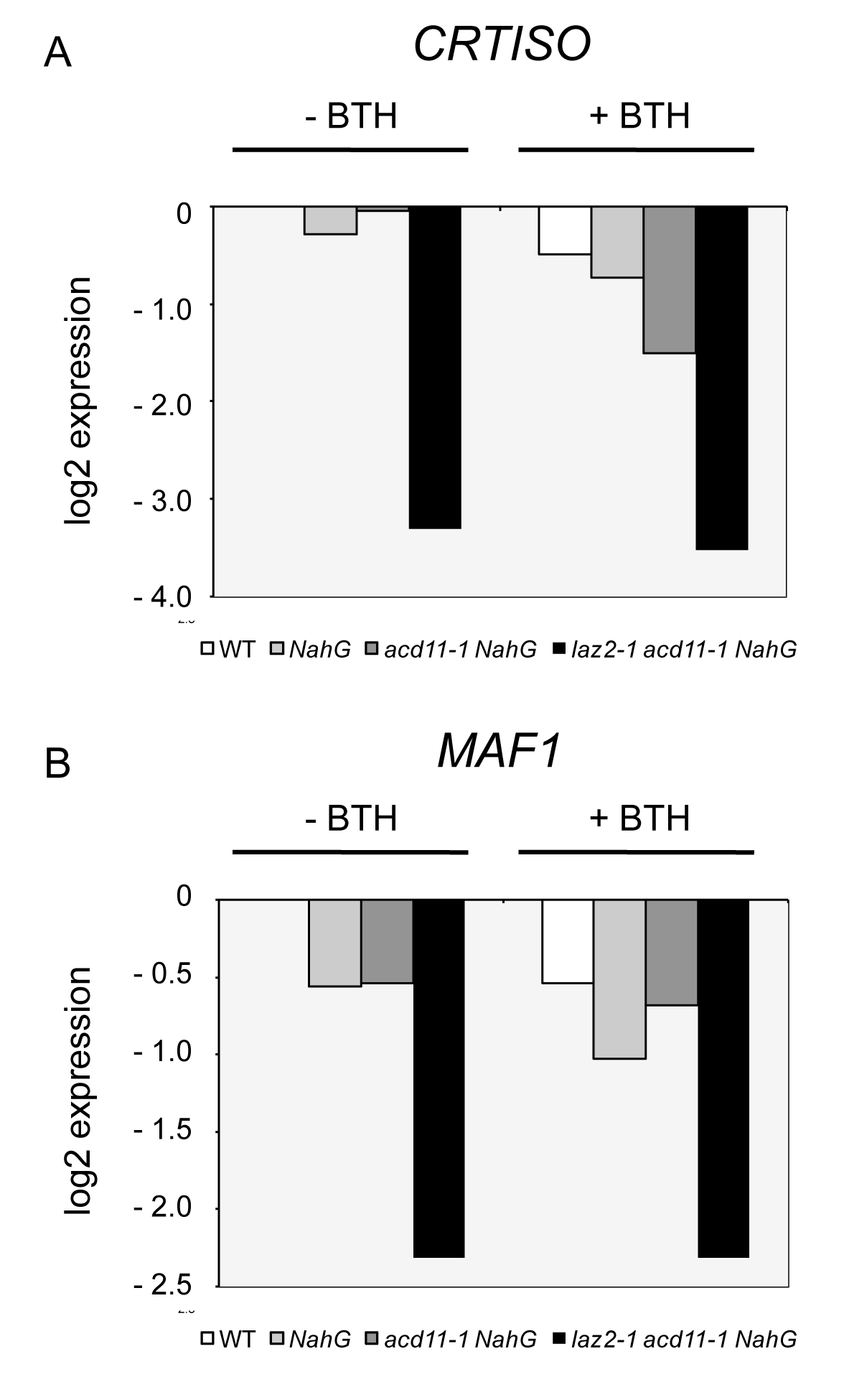

Supplement: Figure S4 — Expression of (A) CRTISO (At1g06820) and (B) MAF1 (At1g77070) in Ler WT, NahG, acd11-1 NahG and laz2-1 acd11-1 NahG before and 72 h after treatment with 100 µM BTH relative to WT at time point 0 (log2 scale). (0.12 MB TIF) [file ppat.1001137.s004.tif]

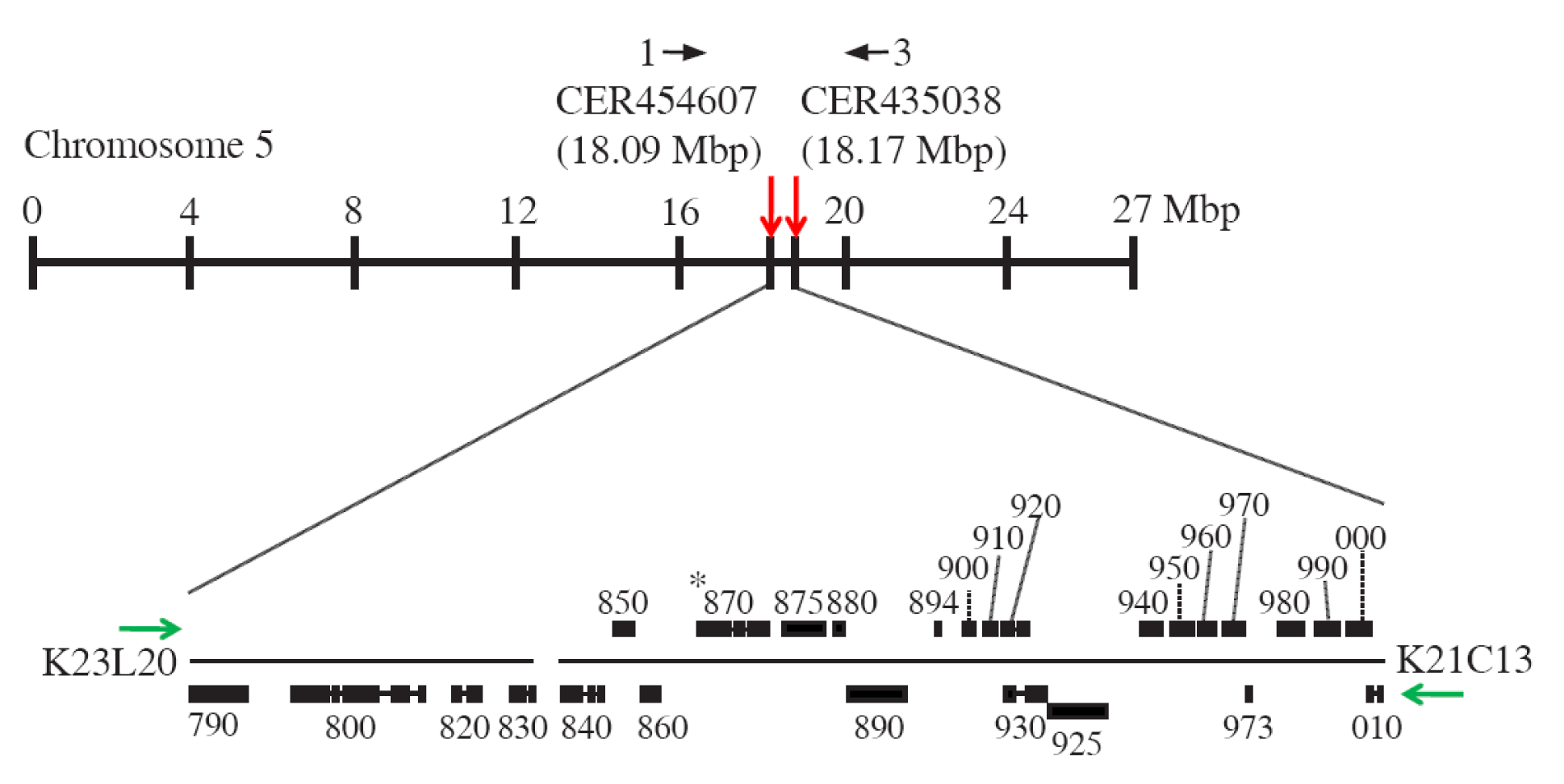

Supplement: Figure S5 — Ecotype-specific markers were used to map the LAZ5 locus to ∼80 kb on the bottom of chromosome 5. Left is centromeric, right is telomeric. Relative positions of mapping markers and numbers of recombinants are indicated. Figure shows a map of the laz5-D1 locus and the genomic region between final recombinants, with associated genes. Asterisk marks the LAZ5 gene with the defect determined by sequencing. (0.24 MB TIF) [file ppat.1001137.s005.tif]

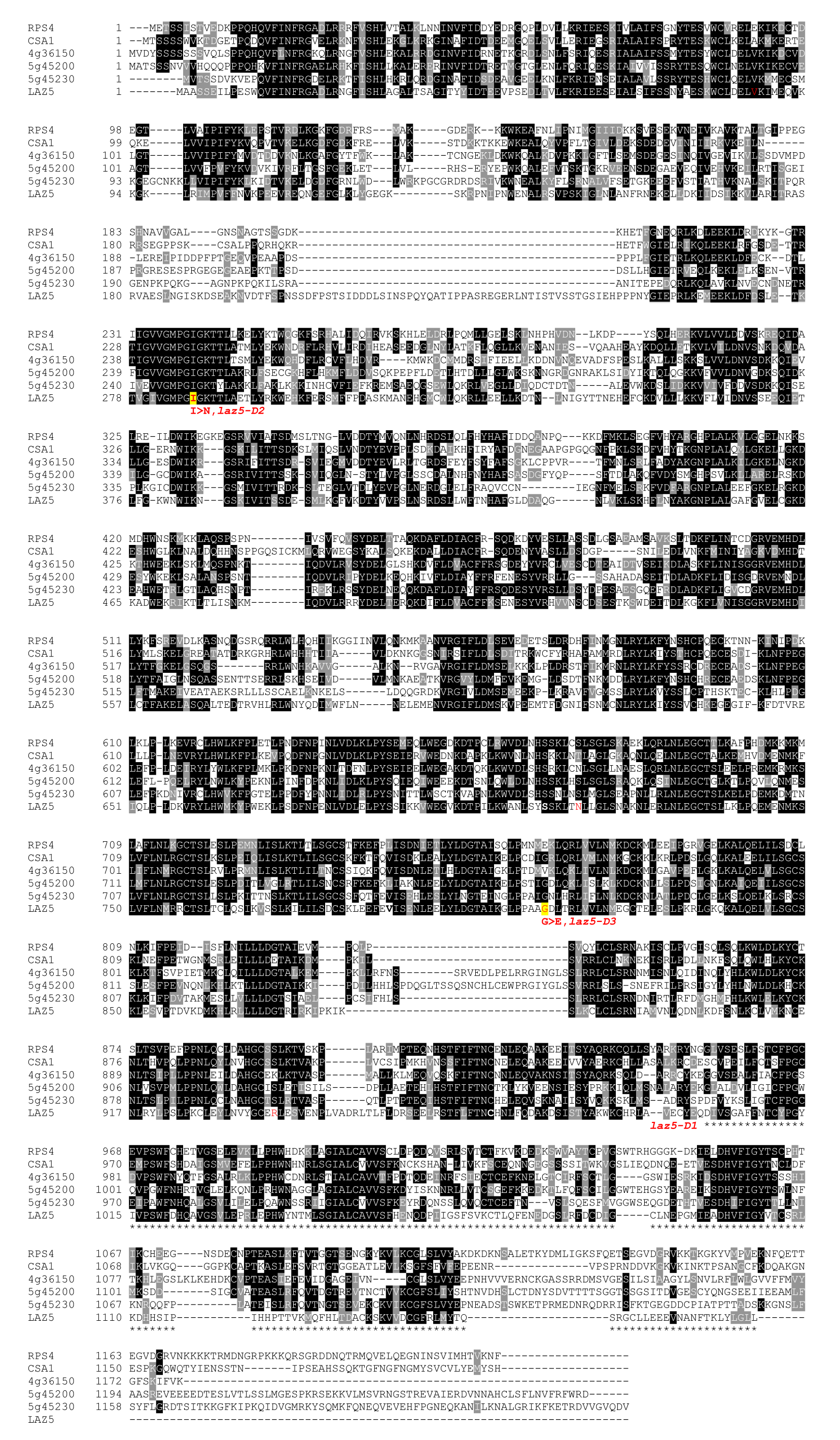

Supplement: Figure S6 — Alignment of LAZ5 and the five most similar Arabidopsis TIR-NB-LRR R proteins, as determined by The Functional and Comparative Genomics of Disease Resistance Gene Homologs Project (http://niblrrs.ucdavis.edu/TN_TNL_phylogeny.html). Sequences include RPS4 (At5g45250), CSA1 (At5g17880), At4g36150, At5g45200, and At5g45230. Mutated residues in laz5-D2 and laz5-D3 are highlighted. Asterisks indicate amino acids predicted to be absent due to the splice site mutation in laz5-D1. (1.35 MB TIF) [file ppat.1001137.s006.tif]

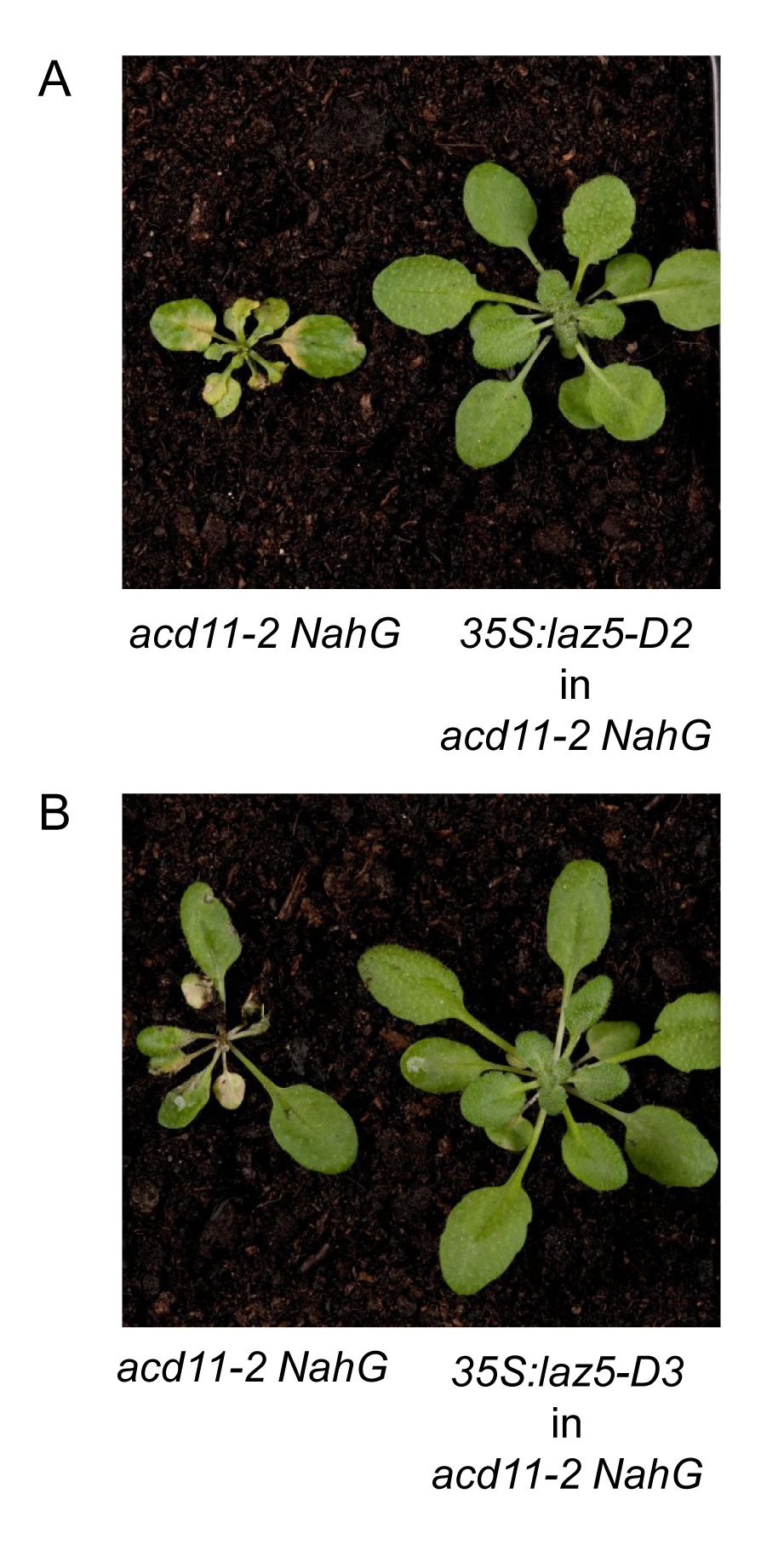

Supplement: Figure S7 — Over-expression of dominant negative laz5-D alleles suppresses acd11. Figure shows acd11-2 NahG (in Col-0) control and representative transgenic lines of acd11-2 NahG stably transformed with (A) 35S:laz5-D2 or (B) 35S:laz5-D3, 10 d after treatment with 100 µM BTH. (1.70 MB TIF) [file ppat.1001137.s007.tif]

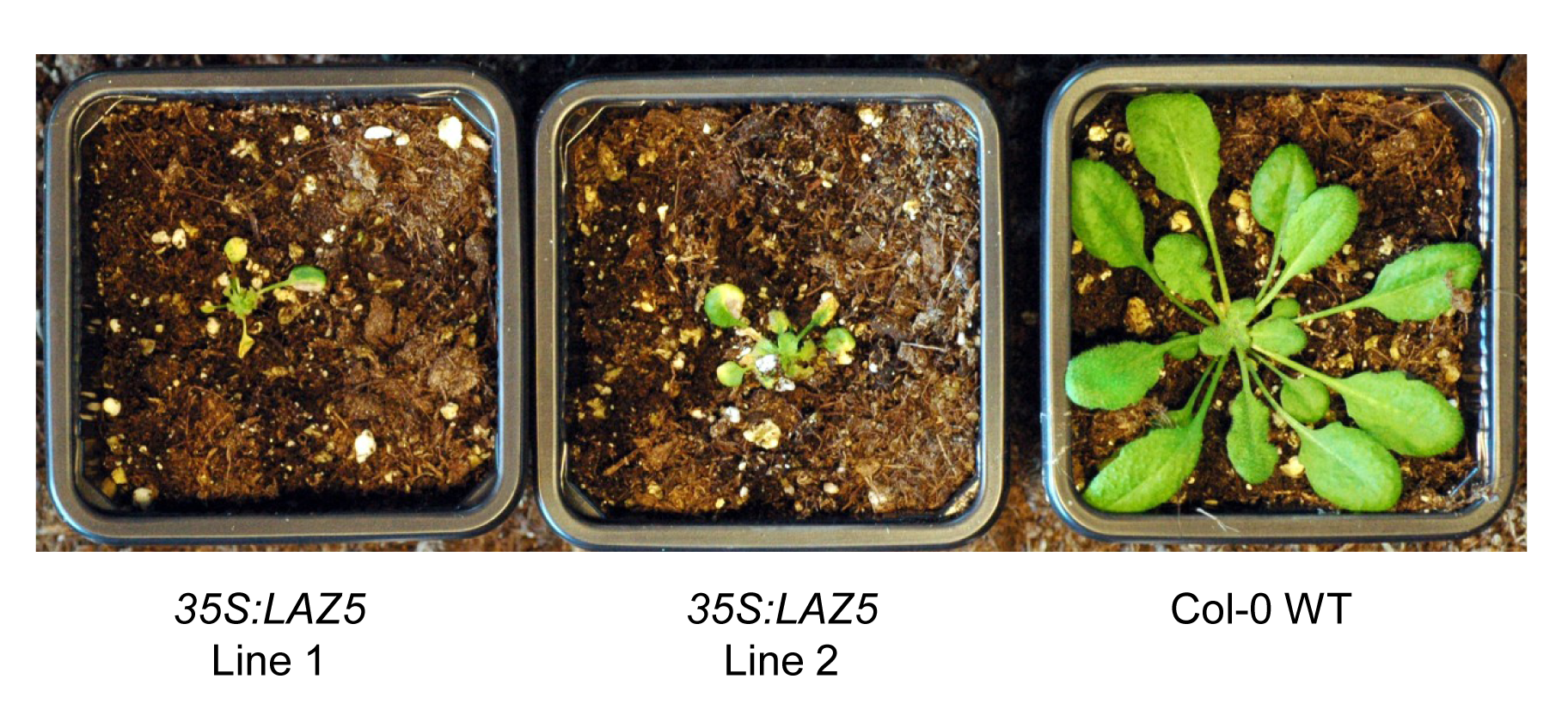

Supplement: Figure S8 — Over-expression of the wild-type LAZ5 R gene results in cell death. Figure shows Col-0 wild-type control and two representative transgenic lines of Col-0 stably transformed with a construct over-expressing genomic LAZ5 (35S:LAZ5). (2.43 MB TIF) [file ppat.1001137.s008.tif]

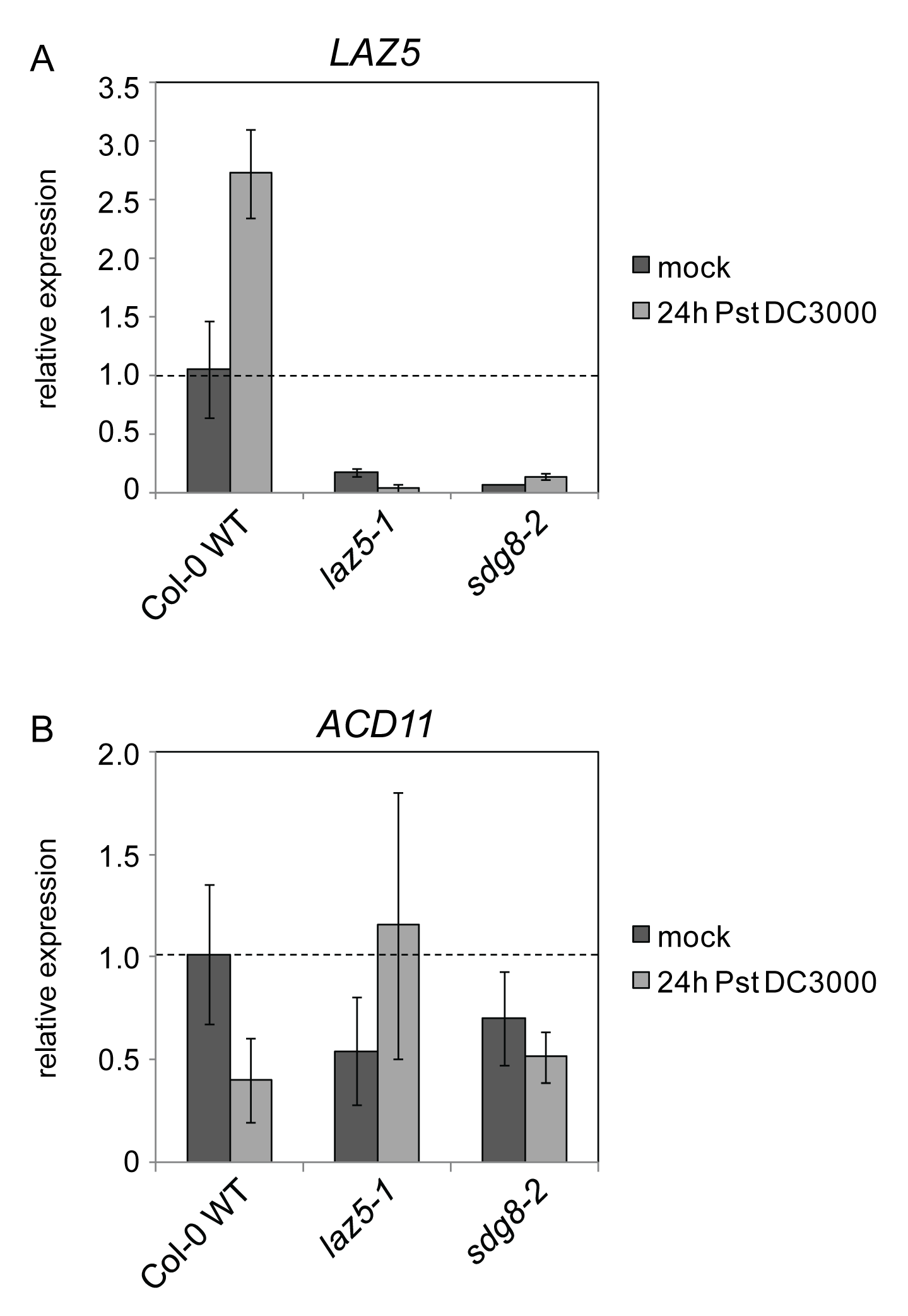

Supplement: Figure S9 — Expression of (A) LAZ5 and (B) ACD11 in 3-week-old Col-0 wild-type, laz5-1 and sdg8-2 mutant plants 24 h after infiltration with P.s.t. DC3000 at OD600 = 0.001 or 10mM MgCl2 mock control, as determined by qRT-PCR. Data is normalized to ACTIN1 (ACT1) and presented as relative expression (fold) compared to Col-0 mock = 1.0 (dashed line), mean ± s.d. (n = 3). (0.15 MB TIF) [file ppat.1001137.s009.tif]

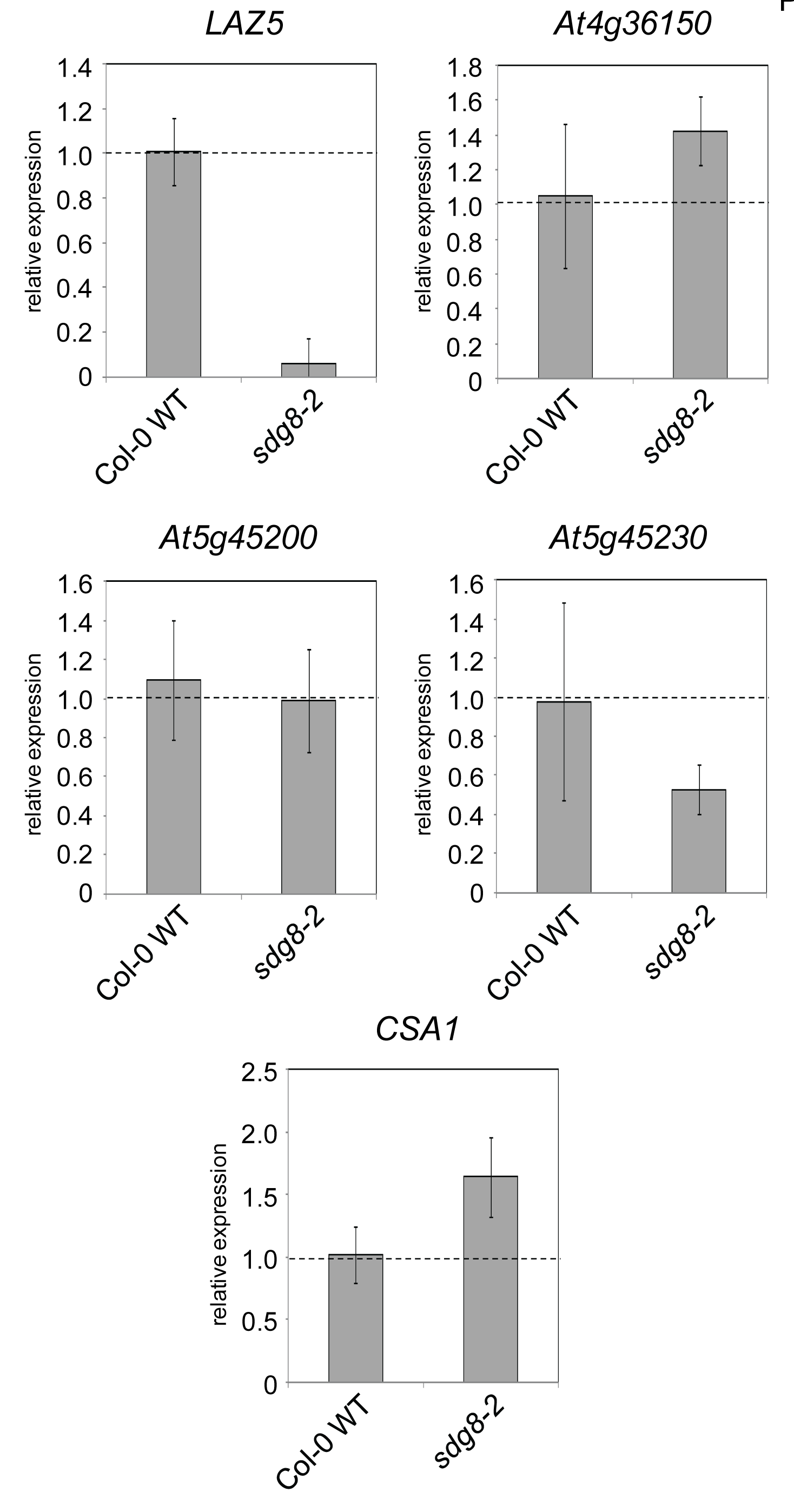

Supplement: Figure S10 — Transcript accumulation of LAZ5 homologs in 3-week-old Col-0 wild-type and sdg8-2 plants, as determined by qRT-PCR. Data is normalized to ACT1 and presented as relative expression compared to Col-0, mean ± s.d. (n = 3). (0.23 MB TIF) [file ppat.1001137.s010.tif]

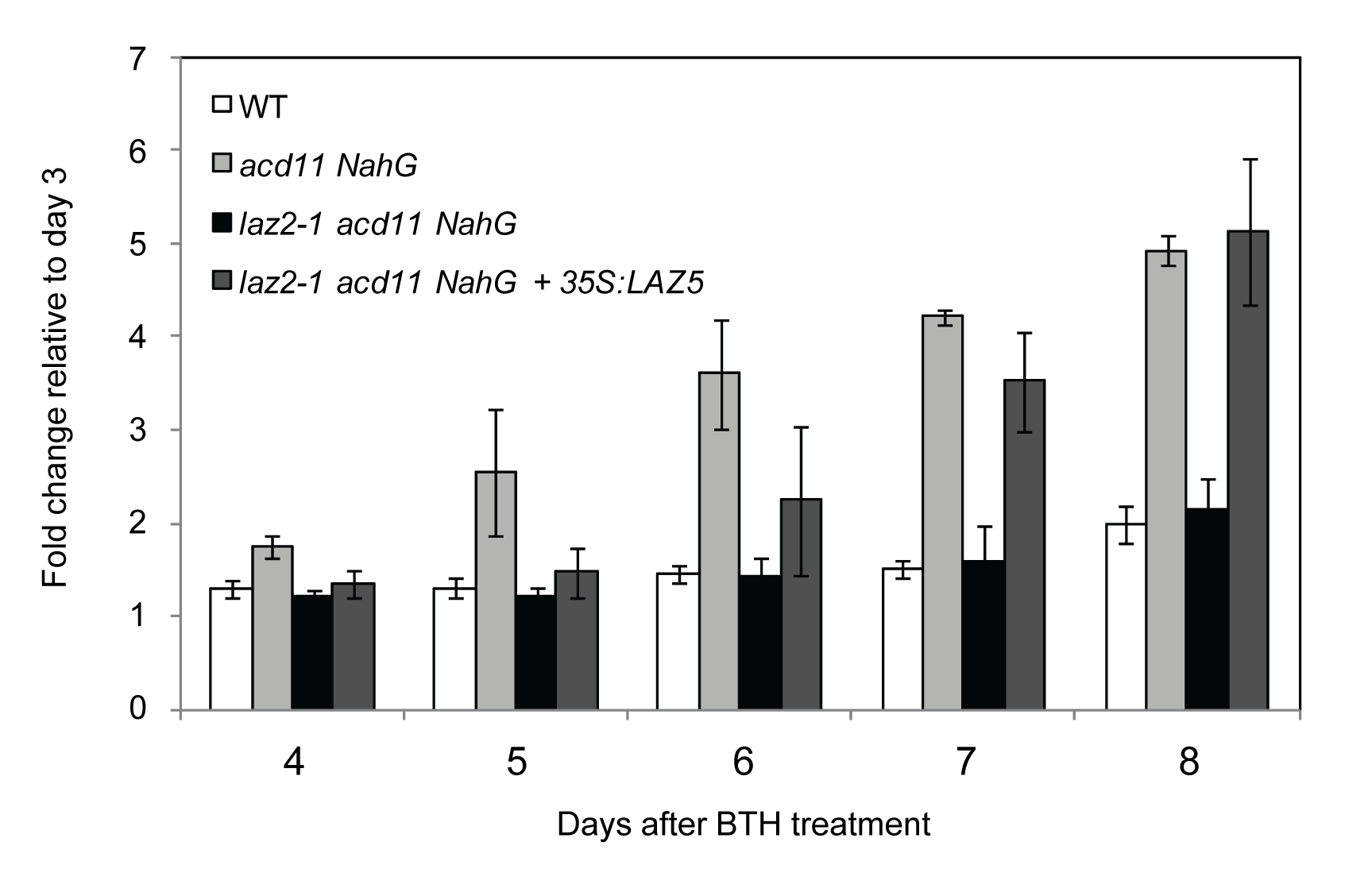

Supplement: Figure S11 — Ion leakage cell death assay of leaf discs from 3-week-old WT, laz2-1 acd11-1 NahG, acd11-1 NahG and laz2-1 acd11-1 NahG over-expressing LAZ5 plants after BTH treatment. The former were selected segregating T2 plants from a transgenic line of genomic LAZ5 in expression vector pGWB521, and confirmed by RT-PCR. Data is presented as fold change in conductivity (µS cm−1) relative to initial value at Day 3. Means ± s.d. were calculated from 6 discs per treatment with 4 replicates within an experiment. (0.13 MB TIF) [file ppat.1001137.s011.tif]

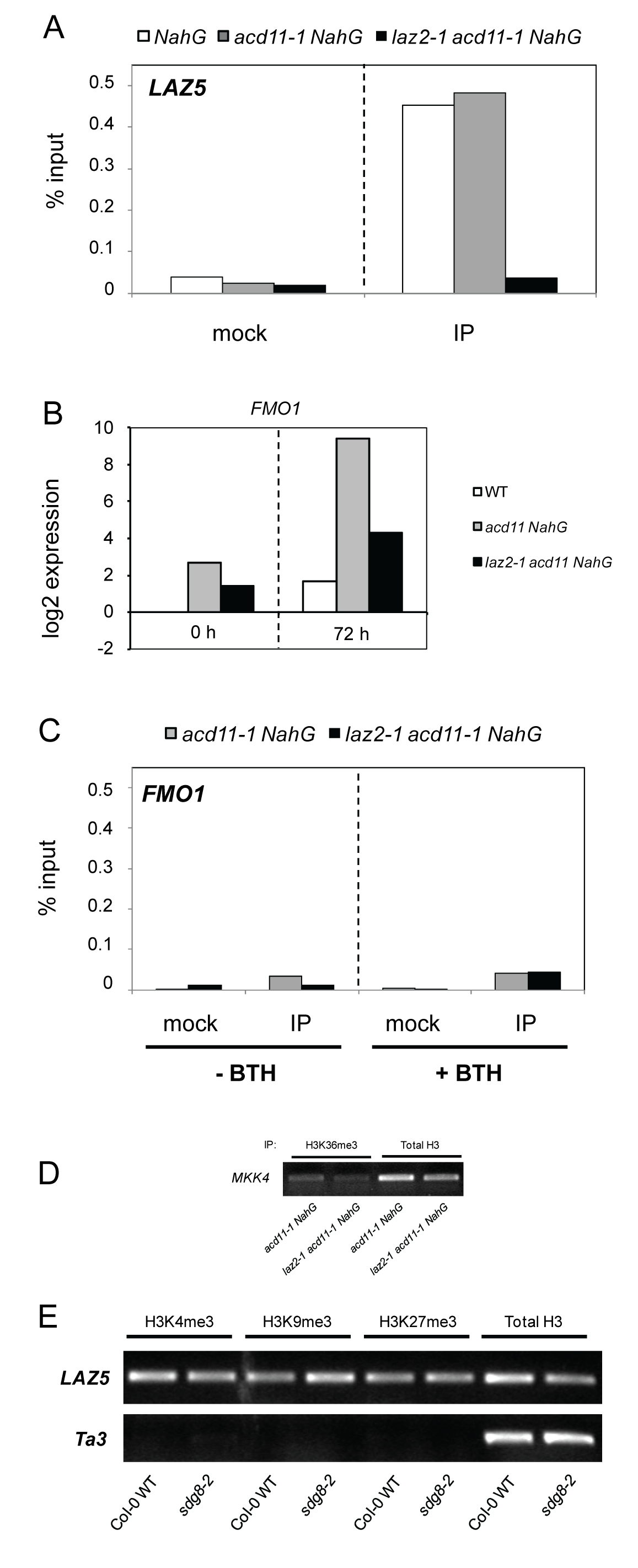

Supplement: Figure S12 — A, H3K36me3 at LAZ5 chromatin is independent of acd11. ChIP analysis of LAZ5 with 1 µg anti-H3K36-me3 antibody (IP) or no Ab (mock) expressed as % input. Tissue was from 3-week-old NahG, acd11-1 NahG and laz2-1 acd11-1 NahG seedlings (Ler background) before and 24 h after treatment with 100 µM BTH. B, H3K36me3 is not a general mark for genes up-regulated in acd11. Expression of FMO1 (At1g19250) in Ler WT, acd11-1 NahG and laz2-1 acd11-1 NahG before and 72 h after treatment with 100 µM BTH relative to wild-type at time point 0 (log2 scale). C, ChIP analysis of FMO1 with 1 µg anti-H3K36-me3 antibody (IP) or no Ab (mock) expressed as % input. Tissue was collected from 3-week-old seedlings. Experiments were repeated twice with similar results. D, H3K36me3 levels at the MKK4 locus is not affected by laz2-1 as determined by ChIP analysis with 1 µg anti-H3K36-me3 antibody or 1 µg anti-H3 (total) antibody, presented as EtBR-stained PCR product (34 cycles). E, Levels of H3K4me3, H3K9me3, H3K27me3 and total H3 at LAZ5 chromatin are not affected by sdg8-2 as determined by ChIP analysis with appropriate antibody. In parallel, ChIP samples were used as templates for PCR at the transcriptionally repressed transposon Ta3 locus for comparison. Data is presented as EtBR-stained PCR product (34 cycles). (0.32 MB TIF) [file ppat.1001137.s012.tif]

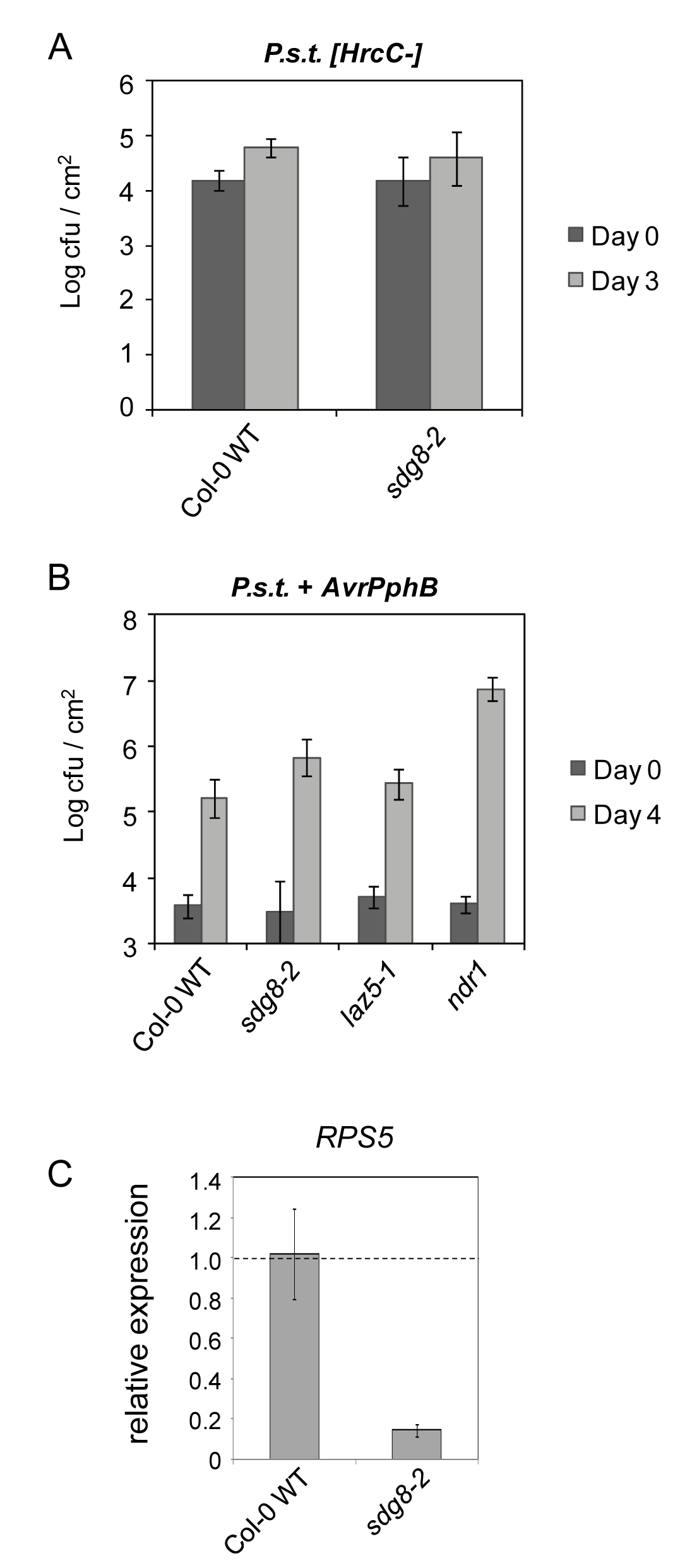

Supplement: Figure S13 — A, Growth of non-pathogenic P.s.t. DC3000 hrcC- mutant in Col-0 WT and sdg8-2 0 and 3 days after infiltration with bacteria at OD600 = 0.001. B, Growth of avirulent P.s.t. DC3000 expressing AvrPphB in Col-0 WT, sdg8-2, laz5-1 and ndr1 plants 0 and 4 days after infiltration with bacteria at OD600 = 0.001. Log-transformed values are means ± s.d. (n = 6). The experiments were repeated once or twice with similar results. cfu = colony forming units. C, Transcript accumulation of RPS5 in 3-week-old Col-0 wild-type and sdg8-2 plants, as determined by qRT-PCR. Data is normalized to ACTIN1 (ACT1) and presented as relative expression compared to Col-0 = 1.0, mean ± s.d. (n = 3). (0.16 MB TIF) [file ppat.1001137.s013.tif]

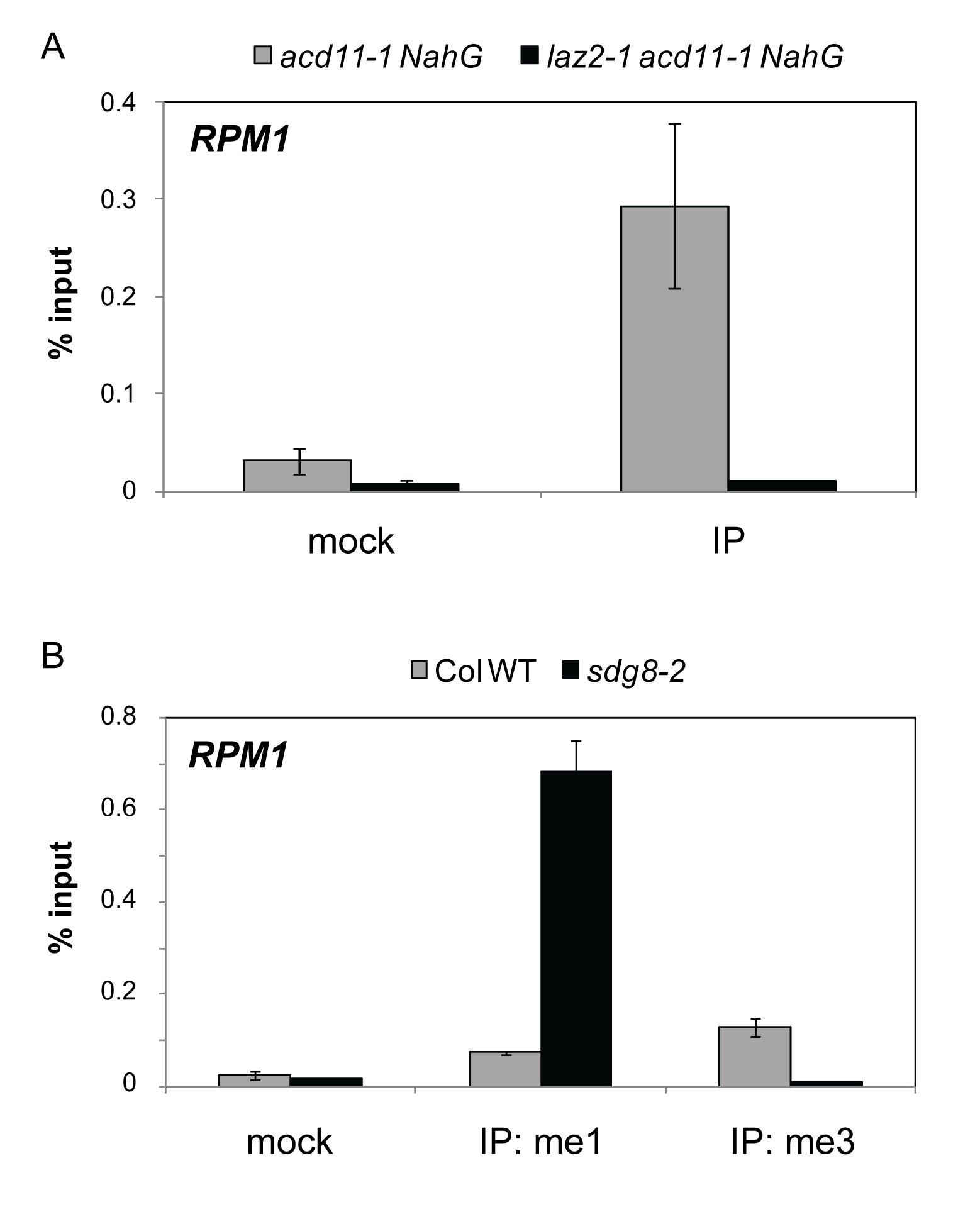

Supplement: Figure S14 — A, ChIP analysis of RPM1 with 1 µg anti-H3K36-me3 antibody (IP) or no Ab (mock) expressed as proportion of input material in the eluate after IP with appropriate Ab (% input), mean ± s.d. (n = 3). Tissue was from 3-week-old acd11-1 NahG and laz2-1 acd11-1 NahG seedlings (Ler background) 72 h after treatment with 100 µM BTH. The experiment was repeated twice with similar results. B, ChIP of RPM1 with 1 µg anti-H3K36-me1 antibody (me1), 1 µg anti-H3K36-me3 antibody (me3), or no Ab (mock) expressed as % input, mean ± s.d. (n = 3). Tissue was from 3-week-old homozygous sdg8-2 and Col-0 WT seedlings. The experiment was repeated with similar results. (0.12 MB TIF) [file ppat.1001137.s014.tif]
